# Identifying glycan consumers in human gut microbiota samples using metabolic labeling coupled with fluorescence-activated cell sorting

**DOI:** 10.1038/s41467-023-36365-8

**Published:** 2023-02-07

**Authors:** Lharbi Dridi, Fernando Altamura, Emmanuel Gonzalez, Olivia Lui, Ryszard Kubinski, Reilly Pidgeon, Adrian Montagut, Jasmine Chong, Jianguo Xia, Corinne F. Maurice, Bastien Castagner

**Affiliations:** 1 Department of Pharmacology & Therapeutics, McGill University, 3655 Prom. Sir-William-Osler, Montreal, Quebec H3G 1Y6 Canada; 2 Canadian Centre for Computational Genomics, McGill Genome Center, 740, Dr. Penfield Avenue, Montreal, Quebec H3A 0G1 Canada; 3 Department of Human Genetics, McGill University, 3640 University, Montreal, Quebec H3A 0C7 Canada; 4 Gerald Bronfman Department of Oncology, McGill University, 5100 Boulevard de Maisonneuve West, Montreal, Quebec H4A 3T2 Canada; 5 Institute of Parasitology, McGill University, 21111 Lakeshore Rd, Ste-Anne-de-Bellevue, Quebec, H9X 3V9 Canada; 6 McGill Centre for Microbiome Research, McGill University, Montreal, Quebec Canada; 7 Department of Microbiology & Immunology, McGill University, 3773 University Street, Montreal, Quebec H3A 2B4 Canada

**Keywords:** Bacteria, Microbiota

## Abstract

The composition and metabolism of the human gut microbiota are strongly influenced by dietary complex glycans, which cause downstream effects on the physiology and health of hosts. Despite recent advances in our understanding of glycan metabolism by human gut bacteria, we still need methods to link glycans to their consuming bacteria. Here, we use a functional assay to identify and isolate gut bacteria from healthy human volunteers that take up different glycans. The method combines metabolic labeling using fluorescent oligosaccharides with fluorescence-activated cell sorting (FACS), followed by amplicon sequencing or culturomics. Our results demonstrate metabolic labeling in various taxa, such as *Prevotella copri*, *Collinsella aerofaciens* and *Blautia wexlerae*. In vitro validation confirms the ability of most, but not all, labeled species to consume the glycan of interest for growth. In parallel, we show that glycan consumers spanning three major phyla can be isolated from cultures of sorted labeled cells. By linking bacteria to the glycans they consume, this approach increases our basic understanding of glycan metabolism by gut bacteria. Going forward, it could be used to provide insight into the mechanism of prebiotic approaches, where glycans are used to manipulate the gut microbiota composition.

## Abstract (web summary)

Dietary glycans are a major driver of the human gut microbiota composition. Here, the authors apply next-generation metabolic labeling coupled with fluorescence-activated cell sorting to identify and isolate gut bacteria consumers of dietary glycans in human stool samples, linking bacteria to the glycans they consume.

## Introduction

The gut microbiota is integral to human physiology, as it metabolizes our diet, synthesizes essential vitamins and amino acids, trains the immune system, and protects us from pathogens^1–5^. Advances in genetic and bioinformatic tools have led to a new understanding of the complexity and diversity of the gut microbiota, as well as its importance in human diseases^6^. The gut microbiome is enriched in genes involved in glycolysis and carbohydrate metabolism^2,7^. Through these carbohydrate-active enzymes (CAZymes), gut bacteria metabolize diet-derived complex glycans (dietary fibers) that reach the colon undigested by the host^8,9^. Consequently, diet is a major determinant of the composition and diversity of gut microbiota, more so than genetic factors^10^. Indeed, changes in diet typically result in rapid microbial metabolic modifications and an altered microbial community structure^7,11^. Importantly, even subtle structural differences in glycans can yield distinct microbial metabolic outputs in human supplementation studies^12^.

Our understanding of CAZymes is rapidly advancing, as exemplified by the characterization of polysaccharide utilization loci (PULs) in Bacteroidetes, such as the starch utilization system (SUS) extensively studied in *Bacteroides thetaiotaomicron*^13^. The *Sus* locus encodes proteins responsible for the binding (SusDEF) and cell surface degradation (SusG) of starch polysaccharides into oligosaccharides that are transported into the periplasm by the TonB-dependent transporter SusC, where they are further processed into monosaccharides by the glycoside hydrolases (GHs) SusAB^14^. Similarly, specific PULs have been described for many other glycans, such as mannan, β-glucan, xyloglucan, and galactomannan^15–19^. On the other hand, Gram-positive bacteria contain transporters that can internalize oligosaccharide structures such as fructooligosaccharides (FOS) by the four-component MsmEFGK in *Lactobacillus acidophilus*^20^, or β-mannans by a solute binding protein (MnBP) and two permeases (MPP) in *Roseburia intestinalis*^21^. Despite these advances, many PULs still have unknown substrate specificities, and GH families can have multiple substrates, making it difficult to predict activity based on sequencing^9,13,22–24^. Thus, functional methods that link glycans with their primary consumers are necessary, especially for Firmicutes and other less studied members of the human gut microbiota^25^.

Several methods have been developed to address this need, with varying levels of complexity, and have been summarized in excellent reviews focusing either on membrane metabolic labeling^26^ or metabolic oligosaccharide engineering^27,28^. As one of the first techniques developed, stable isotope probing (SIP) is a laborious but powerful method to follow metabolism and identify consumers that was applied to starch and galacto-oligosaccharide metabolism in fermentation systems^29–31^. However, SIP generally requires bacterial growth for labeling, thereby inducing bacterial media biases. Recently, Patnode and colleagues used forward genetic screening, quantitative metaproteomics, and artificial food particles to interrogate fiber degradation in mice colonized with synthetic communities of gut bacteria^32^. This approach revealed the contribution of Bacteroides species to the processing of specific glycans and highlighted interspecies competition. More recently, the same researchers used glycan-covered beads to examine the adhesion of bacteria to polysaccharides and revealed strain-level differences in specificity^33^. Notably, they used magnetic beads to recover and identify adherent bacteria from a bacterial coculture and hinted at the possibility of using this method to mine previously uncharacterized consumers^33^. However, it is important to note that these approaches remain to be validated in natural more complex communities.

Next-generation physiology approaches that combine nondestructive cellular phenotyping with separation of cells provide a powerful framework to study microbial physiology and metabolism^27^. Fluorescence-activated cell sorting (FACS) coupled with sequencing (FACSeq) is particularly efficient and can reveal the physiology of individual cells within complex bacterial communities, allowing for the identification of bacteria labeled according to their relative nucleic acid content, cell membrane integrity, and metabolism without prior culturing^34–37^. The FACSeq method can be applied in various contexts such as identifying gut microbiota taxa associated with immunoglobulin A or cholesterol^38,39^. Fluorescent glycans were recently used to demonstrate the uptake of α-mannan oligosaccharides in the periplasm of *B. thetaiotaomicron* by epifluorescence microscopy and FACS^40^, while another study revealed the role of uncultured *Verrucomicrobia* in degrading polysaccharides in aquatic systems using fluorescently labeled glycans, FACS, and single-cell genomics^41^. More recently, Doud and colleagues used fluorescently-labeled cellulose particles, FACS, and amplicon sequencing to uncover cellulose-degrading bacteria in geothermal springs^42^. Collectively, these studies highlight the potential of combining culture-independent approaches to link bacterial diversity to specific metabolism.

Here, we utilized metabolic labeling coupled to FACS and 16S rDNA amplicon sequencing to reveal the bacterial uptake of three structurally different oligosaccharides in stools from three healthy unrelated volunteers. Sorted glycan^+^ cells were enriched in glycan foragers from the Bacteroidetes phylum, but also Firmicutes and Actinobacteria, such as *Blautia wexlerae* and *Collinsella aerofaciens*, respectively. A majority of significantly labeled bacteria were confirmed to be glycan consumers for growth and encoded GHs with activity consistent with the glycans used, yet we were able to identify *B. wexlerea* as a previously unreported FOS consumer. However, three *Bacteroidetes* species labeled by a fluorescent glycan did not show a growth phenotype in vitro with this glycan. In parallel, we isolated bacterial glycan consumers that belonged to three different phyla in a human microbiota sample by culturing glycan^+^ cells. While this method does not label bacteria implicated in cross-feeding, it is a useful method to identify consumers of oligosaccharides.

## Results

### Fluorescent glycan uptake in cultured isolates

**Fig. 1:**
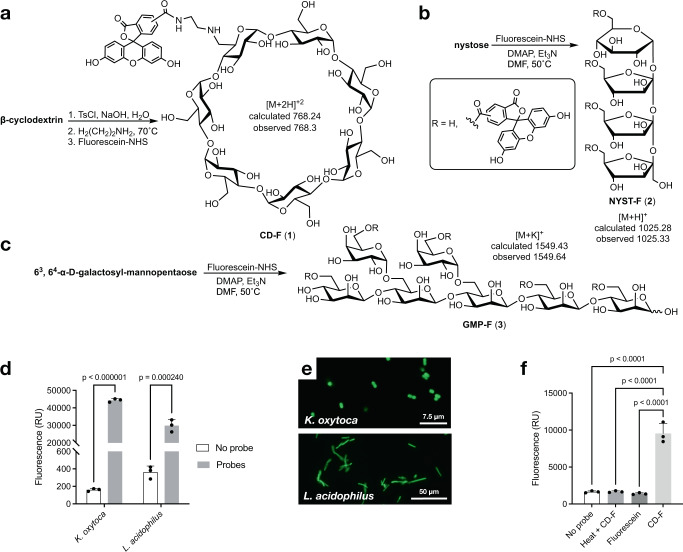
Cultured isolates are metabolically labeled by fluorescent oligosaccharides. **a**–**c** Synthesis of fluorescent β-cyclodextrin (**a**), nystose (**b**), and galactosyl-mannopentaose (**c**) probes. Mono-functionalized glycans were purified by reverse-phase chromatography, size-exclusion chromatography, or high-performance liquid chromatography (HPLC). The products were characterized by mass spectrometry. **d** Fluorescence measured from washed *K. oxytoca* (Proteobacteria) and *L. acidophilus* (Firmicutes) cultures after incubation in their respective minimum media with CD-F or NYST-F probe for 30 min, as measured on a spectrofluorometer. Data are the mean ± SD (*n* = 3). Statistical significance compared to the control condition by unpaired t-tests with Bonferroni-Dunn’s multiple comparison test. **e** Confocal microscopy image of fluorescently labeled *K. oxytoca* and 2-photon microscopy image of *L. acidophilus* after incubation with CD-F and NYST-F. Bacteria in the exponential phase were washed and incubated for 1 h with the probes (500 nM) and then fixed with 4% paraformaldehyde. **f** Fluorescence quantification of bacteria isolated from stool samples after incubation with free fluorescein (4.4 μM) or CD-F (4.4 μM) for 1 h with or without heat shock pretreatment of the gut microbiota for 10 min at 65 °C. Data are the mean ± SD (*n* = 3). Statistical significance compared to the labeled condition by one-way ANOVA with Bonferroni’s multiple comparison test. Source data and statistical details are provided as a Source data file.

To investigate the uptake of fluorescent glycans in gut bacteria, we first synthesized a fluorescein conjugate of β-cyclodextrin, which mimics the helical structure of starch, one of the most common polysaccharides in our diet, and has been used for the structural characterization of *B. thetaiotaomicron* SUS^43^. The monofunctionalized conjugate (CD-F (**1**), Fig. 1a) was purified by preparative HPLC and characterized by mass spectrometry (MS). The specific uptake of CD-F was first investigated in *Klebsiella oxytoca*, a gut bacterium from the Proteobacteria phylum that harbors the CymA transporter specific for cyclodextrin^44^. *K. oxytoca* strain M5A1 was grown in minimum medium M9 (MM M9) supplemented with 0.1% β-cyclodextrin. Bacteria in the log phase that were incubated with CD-F showed a significant fluorescence increase compared to that of the negative control (Fig. 1d), which was corroborated by fluorescence microscopy (Fig. 1e) and flow cytometry (Fig. S1a).

We then prepared fluorescently labeled nystose (NYST-F (**2**), Fig. 1b), a tetrasaccharide derived from inulin, which is a common fructan prebiotic found in wheat, onion, and banana (Fig. 1b)^45^. An unselective functionalization of the nystose hydroxyl groups was performed to obtain a mixture of mono-functionalized nystoses that were conjugated at different positions. This is important since we do not know the putative transporter recognition motif, and a mixture of conjugates is less likely to interfere with binding. Thus, purification of fluorescent nystose (NYST-F) yielded a mixture of products with identical molecular weights that corresponded to the monofunctionalized glycan by mass-spectrometry-coupled liquid chromatography (LC‒MS). Cultures of the known consumer *L. acidophilus* (strain ATCC 4356) were grown in custom De Man, Rogosa, Sharpe (cMRS) broth depleted of glucose and supplemented with inulin, and these cultures were also labeled with NYST-F by fluorescence spectroscopy and microscopy (Fig. 1d, e)^20^.

### Metabolic labeling in the human gut microbiota

**Fig. 2:**
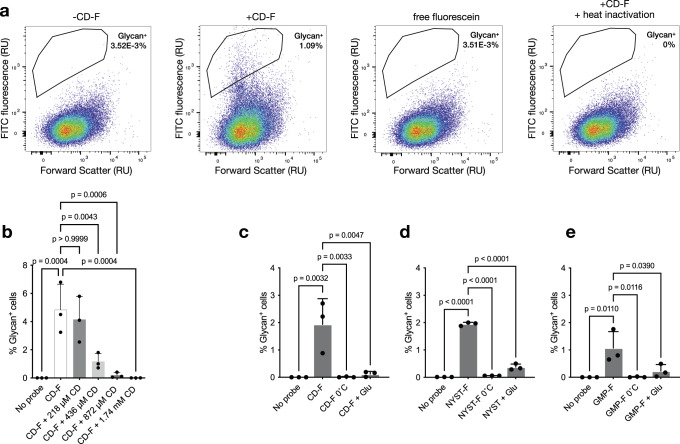
Microbiota metabolic labeling is a specific and energy-dependent process. **a** Representative flow cytometric pseudocolor plots of metabolic labeling. Bacteria isolated from stool were incubated with 4.4 μM CD-F or fluorescein with or without heat inactivation for 1 h, washed, and analyzed by flow cytometry. **b** Quantification of glycan^+^ cells in stool samples incubated with 4.4 µM CD-F probe in the presence of increasing concentrations of free β-cyclodextrin. Bar graph representing means ± SDs (*n* = 3). Statistical significance compared to the CD-F condition by one-way ANOVA with Bonferroni’s multiple comparison test. Specificity of CD-F (**c**), NYST-F (**d**), and GMP-F (**e**) labeling. Bacteria isolated from the stool samples were preincubated in MM at 0 °C or in MM supplemented with 0.1% glucose at 37 °C for 1 h and then labeled with CDF, NYST-F, and GMP-F probes at 4.4 µM for 1 h at 37 °C. After washing, the percentage of glycan^+^ bacteria was measured by flow cytometry. The graphs represent the means ± SDs (*n* = 3) of the percentage of labeled bacteria under different conditions. Statistical significance compared to the CD-F condition by one-way ANOVA with Bonferroni’s multiple comparison test. Source data and statistical details are provided as a Source data file.

After demonstrating the uptake of fluorescent glycans in cultured isolates, we then metabolically labeled bacteria from a stool sample from a healthy human volunteer. Fresh stool samples were placed in anaerobic conditions within 30 min of collection, and aliquots were frozen at −80 °C to be tested further with multiple different glycans. Under these conditions, most bacteria were shown to remain viable^46^. Bacteria were incubated with CD-F for 1 h under anaerobic conditions. Fluorescence measurement of washed cells showed increased fluorescence after incubation with 4.4 μM CD-F for 1 h (Fig. 1f). Importantly, we observed no increase in fluorescence using free fluorescein and when the bacteria were heat-shocked prior to the incubation, suggesting that the fluorescent glycan underwent active transport. Higher concentrations of the fluorescent probe or prolonging the incubation time beyond 1 h did not result in a significant increase in fluorescence (Fig. S1b, c). We then repeated these experiments using flow cytometry to quantify the cell populations that took up the fluorescent glycan. Unlabeled bacteria were used as a negative control to set the threshold of the fluorescent bacterial population. The labeling of a gut microbiota sample with CD-F indicated that ca. 1% of cells were glycan^+^, while no labeling was detected with free fluorescein or after heat inactivation (Fig. 2a).

To demonstrate the specificity of CD-F labeling, we performed a competition experiment with an increasing concentration of unlabeled β-cyclodextrin (CD). Flow cytometry analysis revealed a gradual reduction in labeling that was significant above 436 μM CD, corresponding to a 100-fold excess over CD-F (Fig. 2b). We then sought to verify whether the labeling was due to the uptake of oligosaccharides or if the glycan was hydrolyzed by cell-surface or secreted glycoside hydrolases prior to uptake. We thus synthesized the monosaccharide (glucose-F, **4**) and disaccharide (maltose-F, **5**) equivalent of the CD-F probe (Fig. S2a). Flow cytometry analysis of metabolic labeling showed very little or no labeling with maltose-F and glucose-F compared to CD-F, thus confirming that only larger oligosaccharides with fluorescent labels were taken up (Fig. S2b).

Furthermore, we confirmed that the labeling observed was largely an active, energy-dependent process by performing labeling at 0 °C, which suppressed labeling for both CD-F and NYST-F (Fig. 2c, d). The utilization of complex polysaccharides is also often repressed by the presence of simple monosaccharides, such as glucose^47^. We therefore performed the labeling experiments in minimum media containing 0.1% glucose. Under these catabolic repression conditions, the uptake of our probes was indeed repressed, suggesting a link between probe uptake and metabolism (Fig. 2c, d).

Encouraged by these results, we synthesized a third, structurally different fluorescent oligosaccharide by randomly functionalizing 6^3^,6^4^-α-D-galactosyl-β(1,4)-D-mannopentaose to obtain GMP-F (**3**, Fig. 1c). Galactomannans are hemicellulose glycans found in our diet (e.g., coffee beans and tomatoes), notably in the form of thickening agents, such as guar gum^21^. Metabolic labeling of bacterial cells by GMP-F was confirmed by flow cytometry and was contrasted by a lack of probe uptake at 0 °C and under catabolic repression conditions (Fig. 2e). Taken together, these results suggest that fluorescent oligosaccharides are taken up by gut microbiota members in a specific and energy-dependent fashion, and largely linked to bacterial metabolism.

### Revealing glycan uptake in gut microbiota samples

We then coupled metabolic labeling and FACS with 16S rDNA amplicon sequencing to identify bacteria responsible for glycan uptake. We used our three fluorescent glycans on fecal samples from three unrelated healthy individuals in the same way. Bacteria from stool aliquots were incubated for 1 h with a fluorescent probe in minimum media, washed, and then processed for flow cytometry. Unlabeled control samples were used to set the gating threshold for glycan^+^ cell sorting. Glycan^+^ sorted cells were then lysed before DNA extraction and amplification of the V4 region of the bacterial 16S rRNA gene. Illumina MiSeq sequencing was performed on DNA extracted from 1-3 million sorted cells and on DNA extracted from the initial three stool samples for comparison. Sequence reads were processed and annotated using the ANCHOR pipeline^48^. Briefly, only paired-end sequences in which the primer set was precisely detected were selected, aligned, and dereplicated before selection of exact sequence variants (ESVs). Annotation queried 4 sequence repositories with strict BLASTn criteria (>99% identity and coverage). Note that all annotations are considered putative and subject to improvement as database errors are resolved and new species are characterized.

We obtained an average of 68,122 read counts per sample, ranging from 29,360 to 85,124. The ANCHOR pipeline identified a total of 334 distinct ESVs. Of these, 93 ESVs could be unambiguously assigned to a species with 100% coverage and >99% identity, and 100% identity was obtained for 86 out of the 93 ESVs. The sorted glycan^+^ samples exhibited lower bacterial α-diversity indices (observed ESVs, Chao1, and Shannon) than those of the starting stool samples, which is consistent with the labeled cells representing a subset of the initial gut microbiota samples (Fig. S3a). A principal component analysis (PCoA) showed a clustering of samples according to the individual (Fig. S3b), indicating that interpersonal differences in microbial communities explained most of the variance (45%). However, constrained ordination (CAP) analysis was performed using the labeled cells versus the initial stool samples as an a priori hypothesis and produced two distinct clusters on an ordination axis; this result explained 9% of the variance (Fig. S3c) and once again supported the enrichment of specific bacterial taxa from the original stool sample. Clustering of NYST-F^+^ cells from the other two probes was observed by further constraining the analysis by glycan type (Fig. S3d).

**Fig. 3:**
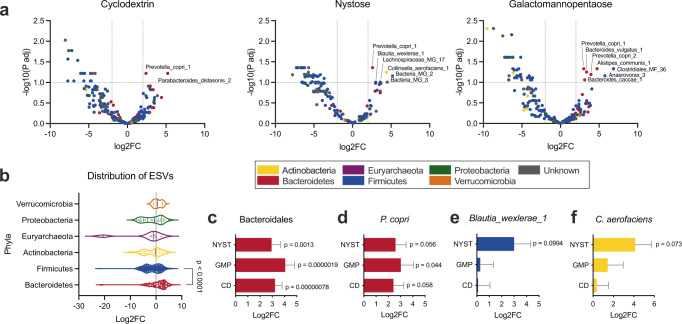
Metabolic labeling enriches different bacteria depending on the glycans applied. **a** DESeq2 differential abundance analysis comparing the ESV abundance in the starting stool versus the glycan^+^ samples expressed as a volcano plot of the adjusted *p* value as a function of the log2-fold change. The horizontal line represents statistical significance and is drawn at an FDR of 0.1. The symbols are colored according to the phyla, and the ESVs that are statistically overrepresented in the glycan^+^ samples are labeled. **b** Distribution of the ESVs from the main phyla on the differential abundance axis. Statistical significance compared the Bacteroidetes and the Firmicutes by a two-tailed Mann‒Whitney test. **c**–**f** DESeq2 differential abundance bar graphs for the Bacteroidales order (**c**), *Prevotella copri* (**d**), *Blautia wexlerae_1* (**e**), and *Collinsella aerofaciens* (**f**). Bar graphs represent log2 fold change ± log2 fold change standard error determined by DESeq2. Source data and statistical details are provided as a Source data file.

A DESeq2 differential abundance analysis was performed to identify ESVs that were overrepresented in the CD-F^+^, GAL-F^+^, or NYST-F^+^ samples compared to the initial stool samples using a false discovery rate (FDR) of 0.1 as a threshold for significance^49^. Despite the low number of samples, multiple ESVs increased above significance and were clearly overrepresented in the glycan^+^ samples (Fig. 3a). Shotgun metagenomic sequencing of one of the starting stool samples was performed in triplicate, and matching sequences were found for each of the statistically significant ESVs, confirming their identity (Supplementary Table 2). Furthermore, since ANCHOR retained 16S sequences, genomes of the significant bacterial species could be matched with 100% coverage and identity for 7/8 of the statistically significant ESVs; thus, we could analyze their genomes more extensively (Supplementary Table 3).

ESVs belonging to the Bacteroidetes phylum were overrepresented in glycan^+^ samples compared to Firmicutes (Fig. 3b). Furthermore, the Bacteroidales order was significantly overrepresented in the glycan^+^ labeled samples for all glycans, indicative of the general ability of its members to metabolize glycans (Fig. 3c)^8^.

Overall, we found nine ESVs identified at the species level that were overrepresented in the glycan^+^ samples (Fig. 3a). Of these, four were known based on previous metabolism ability. *Prevotella copri* (Bacteroidetes) was found to be overrepresented in all three glycan^+^ samples (Fig. 3d), which supports its extensive repertoire of polysaccharide utilization genes and known ability to grow on starch, inulin, and galactomannan^50,51^. In addition, we observed that *Parabacteroides distasonis* was labeled by cyclodextrin (Fig. 3a). Our 16S sequence matched with 100% homology and coverage for the *P. distasonis* CL03T12C09 strain, which contains 6 glycoside hydrolases family 13 (GH13, α-amylase) in its CAZyme repertoire that are consistent with the metabolism of starches.

The following ESVs were overrepresented in the NYST^+^ samples and were not known FOS consumers: *Blautia wexlerae* (Firmicutes) and *Collinsella aerofaciens* (Actinobacteria) (Fig. 3a, e, f). Furthermore, the following Bacteroidales that were overrepresented in the GMP^+^ samples were not known galactomannan consumers: *Alistipes communis*, *Bacteroides vulgatus*, and *Bacteroides caccae* (Fig. 3a). We therefore examined these interactions in more detail to determine if these glycans lead to growth of these isolates in vitro.

**Fig. 4:**
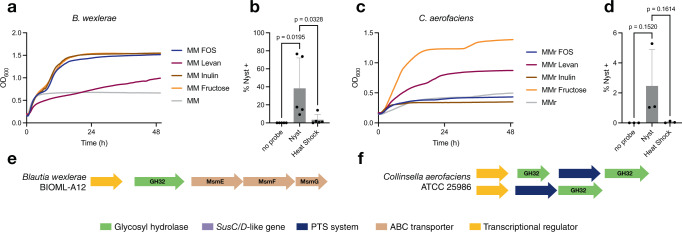
*Blautia wexlerae* and *Collinsella aerofaciens* are FOS and levan consumers, respectively. Growth of *B. wexlerae* (**a**) and *C. aerofaciens* (**c**) in MM supplemented with different fructooligosaccharides for 48 h. The viability of bacteria was evaluated by growth in MM supplemented with fructose. All growth measurements are the means of triplicates. Exponential cultures of *B. wexlerae* (*n* = 5) (**b**) and *C. aerofaciens* (*n* = 3) (**d**) in MM supplemented with FOS were incubated for 1 h with NYST-F. The percentage of NYST^+^ bacteria was measured by flow cytometry. Data from three to five independent experiments are shown as the means ± SD. Statistical significance compared to the GMP-F condition by one-way ANOVA with Bonferroni’s multiple comparison test. Map of potential FOS metabolism-associated genes. Identification of putative genes involved in the metabolism of FOS in *B. wexlerae* (**e**) and *C. aerofaci****e****ns* (**f**). BLAST alignments were performed on the complete genome available in the NCBI database with the known glycoside hydrolases that are involved in GMP and FOS metabolism. Source data and statistical details are provided as a Source data file.

*B. wexlerae* was shown to be increased in a pig FOS supplementation study^52^ and in human microbiota ex vivo fermentation^53^, but little is known about its glycan metabolism, and this species is not present in the CAZy database^24^. As the genetic basis of FOS metabolism in *L. acidophilus* involves a MsmEFGK ABC transporter and a GH32^54^, we searched for this homologous PUL in *B. wexlerae* strain BIOML-A12, which matched our sequence with 100% identity and coverage. We found a similar system in *B. wexlerae* that contained GH32 and MsmEFG transporter genes, thus supporting FOS uptake and metabolism (Figs. 4e, S4a). We then obtained *B. wexlerae* (DSM 19850, 100% identity and coverage to our ESV) and evaluated its ability to grow on various carbohydrates. While the strain exhibited minimal growth in minimum media depleted of carbohydrates, it showed robust growth when fructose, FOS, or inulin was supplemented in the medium (Fig. 4a). Furthermore, only slow growth was observed with levan, a related β-2,6-fructo-oligosaccharide. In addition, *B. wexlerae* in culture was robustly labeled by NYST-F (Fig. 4b). Therefore, *B. wexlerae* is a FOS consumer and contains putative genes consistent with FOS metabolism for growth.

We then examined *C. aerofaciens*, which was reported to exhibit a weak growth phenotype on levan but not FOS^55^. In the CAZy database, three GH32s are annotated in the genome of *C. aerofaciens* ATCC 25986 (100% identity and coverage to our ESV). The first PUL comprises two GH32 genes flanking a phosphoenolpyruvate:sugar phosphotransferase system (PTS) subunit IIC gene. The second locus contains a GH32 and a PTS subunit IIB gene. These two loci are homologous to known PULs involved in FOS metabolism in the closely related *Anaerostipes hadrus*, suggesting that they are involved in fructose polymer metabolism (Figs. 4f, S4b)^56^. We obtained *C. aerofaciens* ATCC 25986 and confirmed that it could grow slowly in minimum media containing yeast and beef extract supplemented with levan, but not FOS or inulin (Fig. 4c). Nevertheless, *C. aerofaciens* isolated cultures were labeled by NYST-F (Fig. 4d). This result shows that while *C. aerofaciens* consumes oligofructose, it is specific for the β-2,6 linkages found in levan, as opposed to the β-2,1 linkage in FOS and inulin.

*B. vulgatus* and *B. caccae* were also significantly labeled by GMP-F (Fig. 3a) despite a lack of GH26 in the genomes of sequenced isolates in the CAZy database^24^. In addition, *B. caccae* is known to not grow on this substrate^55^. We thus cultured *B. vulgatus* (ATCC 8482) and *B. caccae* CLD22004 (which was isolated from one of the fecal samples, *vide infra*) and confirmed the lack of growth in minimum media (MM) with 0.1% GMP as a sole carbon source, whereas growth was observed in MM supplemented with glucose (Fig. S5). We further confirmed that both strains were labeled by GMP-F after growth in BHI, as seen by flow cytometry (2.18% and 1.25% of glycan^+^ cells for *B. vulgatus* and *B. caccae*, respectively), which was abrogated by heat shock (Fig. S5). These data suggest that some fluorescent glycan uptake took place, but probably by a PUL with a related substrate, or that the metabolism was not sufficient to sustain the growth of these isolates.

Similarly, *Alistipes communis* (a.k.a. *Alistipes obesi*)^57^ was labeled by galactomannopentaose (Fig. 3a). Little is known about galactomannan metabolism in *A. communis*, but this bacterium is known to exhibit α-galactosidase activity^58^. The *A. communis* 5CBH24 strain (100% identity and coverage to our ESV) encodes a classic SusC/D-like-containing PUL with 2 GH26s (with β1-4-mannanase activity), a GH130 (a mannosylphosphorylase), and a GH97 (α-galactosidase activity), which is consistent with galactomannan metabolism (Fig S5j) and resembles the known galactomannan PUL in *B. ovatus*^18^. However, while *A. communis* 5CBH24, could grow slowly in Columbia media depleted with carbohydrate, its growth was increased by supplementing glucose but not galactomannan (Fig. S5g), suggesting that this strain is incapable of utilizing this substrate for growth.

Taken together, these results demonstrate that 66% (6/9) of the ESVs significantly overexpressed in the glycan^+^ samples were known or confirmed consumers of the glycan, but that 33% (3/9) could not use the glycans for growth in vitro.

### Isolation of glycan consumers from stool samples

**Fig. 5:**
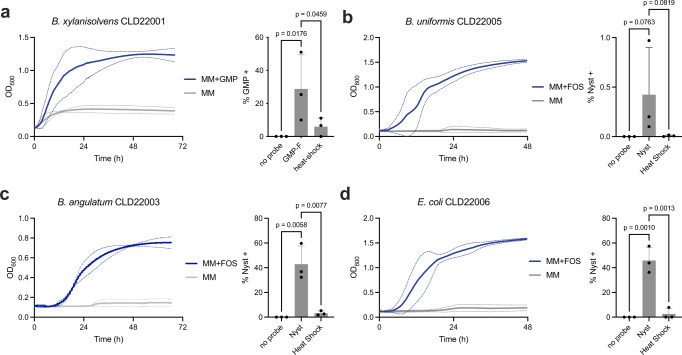
Consumer bacteria from three major phyla can be isolated by culturing glycan^+^ cells. **a** Growth of *B. xylanisolvens* CLD22001 in minimum medium supplemented or not with 0.1% galactomannopentaose. The growth curves are the mean and standard error of three independent replicates. GMP-F uptake by *B. xylanisolvens* CLD22001. The exponential cultures of *B. xylanisolvens* CLD22001 in MM supplemented with GMP were incubated for 1 h with GMP-F. The percentage of GMP^+^ bacteria was measured by flow cytometry. Data from three independent experiments are shown as the means ± SD. Statistical significance compared to the GMP-F condition by one-way ANOVA with Bonferroni’s multiple comparison test. Growth of *B. uniformis* CLD22005 (**b**), *B. angulatum* CLD22003 (**c**), and *E. coli* CLD22006 (**d**) on MM supplemented or not with 0.1% FOS. Quantification of NYST-F uptake by *B. uniformis* CLD22005 (**b**), *B. angulatum* CLD22003 (**c**), and *E. coli* CLD22006 (**d**) by flow cytometry, with or without heat shock before labeling. All measurements were performed in triplicate, and the mean ± SD are displayed. Statistical significance compared to the probe condition by one-way ANOVA with Bonferroni’s multiple comparison test. Source data and statistical details are provided as a Source data file.

Considering that glycan consumers were enriched in the sorted labeled cells, we further used this subset of cells to isolate specific glycan consumers from human fecal samples. This gave us access to the bacterial strains present in the sample and allowed us to study their growth phenotype directly. First, fecal bacteria were labeled with GMP-F for 1 h, and glycan^+^ cells were sorted for 5 min (reaching approximately 27k sorted cells) before immediately being resuspended in reduced PBS in anaerobic conditions. Diluted bacteria were then cultured on BHI for 72 h. A GMP consumer (strain CLD22001) was isolated from this culture, as demonstrated by its ability to grow in MM with 0.1% GMP as a sole carbon source but not MM alone (Fig. 5a). We then performed DNA extraction and amplification of the whole 16-S rDNA region followed by Sanger sequencing. By performing BLAST alignment of the V1V9 sequence to the NCBI nucleotide database collection, strain CLD22001 was identified as a *Bacteroides xylanisolvens* with 99.79% identity and 100% coverage with *B. xylanisolvens* (strain funn3). Multiple members of the *B. xylanisolvens* harbor the galactomannan PUL that was characterized in *B. ovatus*, allowing them to grow on galactomannan^18,19^. By flow cytometry, we confirmed that *B. xylanisolvens* CLD22001 cells were labeled by GMP-F when grown on MM containing GMP (Fig. 5a). In the same way, we isolated *B. caccae* strain CLD22004, that has 100% coverage and 98.95% identity with strain CL03T12C61. We demonstrated that this strain was labeled with GMP-F but could not grow on GMP (Fig. S5a–c).

We then cultured NYST-F^+^ sorted cells to isolate FOS consumers. Here, the sorted labeled bacteria were immediately resuspended in reduced ABB medium supplemented with 0.1% FOS under anaerobic conditions. After 48 h of incubation, the cultured bacteria were transferred in a dilution of 1/100 into MM supplemented with 0.1% FOS as the sole carbon source and incubated for 48 h under anaerobic conditions. The FOS consumers were then isolated on ABB or TSA supplemented with 5% sheep blood for 72 h and then characterized as described above. We isolated a *Bacteroides uniformis* (strain CLD22005, with 100% coverage and identity with strain CL11T00C07) that could grow in MM containing FOS but not MM alone (Fig. 5b). The labeling of cultured isolates was low but abolished by heat shock (Fig. 5b). *B. uniformis* (CL11T00C07) contains a predicted PUL 32 containing 4 GH32s; a GH172; and a carbohydrate binding module family 38 (CBM38), which is known for inulin binding, along with the SusCD transporter, and these results are consistent with FOS consumption. Consequently, *B. uniformis* is known to grow on fructose and inulin^59^. In addition, strain CLD22003 that grew on MM supplemented with FOS was identified as *Bifidobacterium angulatum* (Actinobacteria) with 100% coverage and 99.79% identity with multiple strains including DMS 20098. The FOS metabolism of *B. angulatum* was confirmed by growth on MM supplemented with FOS as a sole carbohydrate source and a strong uptake of the NYST-F probe, as measured by cytometry (Fig. 5c). The genus *Bifidobacterium* is well-known for FOS metabolism in studies involving diets enriched by FOS supplementation, and *B. angulatum* is a known consumer in vitro^60,61^. Finally, we obtained a few Proteobacteria, including strain CLD22006 with a 16-S gene matching several *Escherichia coli* strains with 100% coverage and identity, including strains TUM13867. The *E. coli* strain could grow in MM containing FOS, and NYST-F labeling was robust (Fig. 5d). Enterobacteria, such as *E. coli*, have been reported to metabolize FOS^62^.

These results demonstrate that functional sorting and culturing can lead to the isolation and identification of glycan consumers from stool samples. While we investigated three different glycan structures in detail here, the method is applicable to other structures. Indeed, we expanded our glycan repertoire by preparing 5 additional fluorescein-conjugated oligosaccharides based on xylan, mannan, arabinoxylan, arabinan, and FOS (Fig. S6a) and confirmed by flow cytometry that these additional glycans were taken up by bacteria in a fecal sample (Fig. S6b).

## Discussion

As we progress beyond a descriptive understanding of the human gut microbiota, functional assays are necessary to examine the role of specific bacteria within the community and in relation to the host^25^. Diet-derived complex glycans are a major driver of the diversity and metabolism of gut microbes, but we still have an incomplete understanding of specific glycan metabolism in the gut. However, this knowledge is crucial for understanding the mechanisms of prebiotic applications, in which microbiota-accessible glycans are used to manipulate gut microbial composition and metabolism^63–66^. Identifying gut bacteria that consume prebiotic glycans is an important step to ultimately reveal bacterial factors or metabolites that mediate host health benefits^67^.

Here, we used a functional assay that combined active metabolic labeling with FACSeq or culturomics to shed light on glycan uptake in human gut microbiota samples, without prior knowledge of the metabolic genes involved. Metabolic labeling of bacteria by fluorescent glycans is possible because oligosaccharides are taken up into bacteria, allowing a small fluorescent tag to go unnoticed^15,40^. Indeed, the prototypical SUS hydrolyzes polysaccharides at the cell surface into oligosaccharides that are taken up within the cell for further metabolism. This uptake prevents other bacteria from benefiting from any metabolic byproducts. Our experiments showed that the glucose monosaccharide probe was not taken up in contrast to larger oligosaccharides, which were transported into bacterial cells. This suggests that the fluorescein tag interferes with the transport of small monosaccharides but not larger oligosaccharides. However, it is also possible that ring opening or partial hydrolysis of cyclodextrin occurs prior to intracellular uptake. We minimized the potential detrimental effects of dye conjugation by random functionalization at different positions, thus increasing the chances that the glycan recognition motif of the carbohydrate-binding domain or transporter was not masked by the fluorophore. In contrast with selfish polysaccharide utilization, some gut bacteria hydrolyze polysaccharides at the cell surface, releasing monosaccharides that are readily utilized by other species^68^. Our approach only detects oligosaccharide uptake and does not identify bacterial cross feeding or downstream secondary metabolism, which remain to be determined. In addition, it is possible for an oligosaccharide to be partially hydrolyzed by one bacterium and taken up by another^68,69^.

This proof-of-principle study was performed using three different glycans on samples from three unrelated healthy volunteers. The differential abundance analysis highlighted the uptake ability of several bacteria, and some of these bacteria were already known to consume starch, galactomannan, and FOS. Our work clearly highlighted the generalist ability of the Bacteroidales order, in line with their known large CAZyme repertoire, while glycan uptake in the Firmicutes was more sparsely distributed and limited to only one of the glycans tested, again consistent with the fact that they have a smaller repertoire of CAZymes than Bacteroidetes^8^.

Importantly, this pipeline allowed us to identify Firmicutes *B. wexlerae* as a consumer of FOS and inulin. This metabolism is likely mediated by a PUL containing a GH32 and an ABC transporter with homology to the FOS PUL of *L. acidophilus* (Fig. S4b). Expanding the screening to different glycans and different samples will likely yield other previously unknown consumers. We have already demonstrated the synthesis of 5 additional probes and demonstrated their labeling of gut bacteria in one fecal sample (Fig. S6), highlighting the feasibility of our approach.

The labeling observed here was largely abrogated at 0 °C or in the presence of glucose, suggesting an active process linked to metabolism. However, three bacteria (*A. communis*, *B. vulgatus*, and *B. caccae*) labeled by GMP-F lacked the ability to grow on galactomannan in vitro. While GMP-F may bind to surface proteins without uptake in this case, the loss in fluorescent signal upon temperature inactivation supports the active uptake of glycans. It is possible that the glycan activates and is bound to or taken up by an active PUL on a structurally related glycan and that GH specificity precludes efficient metabolism. Indeed, *C. aerofaciens* was labeled by nystose containing β-2,1 fructose linkages, but could not grow on this substrate and instead metabolized levan containing β-2,6-fructose linkages for growth. Furthermore, *A. communis* harbors a PUL with a strong resemblance to the known galactomannan PUL of *B. ovatus*, suggesting activity on an α-galactose- and β-mannose-containing substrate, even if growth on galactomannan was not observed^18^. The absence of growth in bacterial strains encoding the appropriate PUL in their genome has been observed for arabinogalactan in *P. copri* and may be due to slight differences in substrate or inaccurate specificity based on gene annotation^50^. Alternatively, glycans can be recognized by proteins implicated in quorum-sensing pathways or host sensing pathways, as has been suggested for fucose in pathogens^70,71^. Therefore, metabolic labeling alone, even with genes consistent with an activity, is not sufficient to infer metabolism for growth, and further studies are needed to fully characterize the possible metabolic pathways involved and their function for the bacterial cell.

We therefore expanded our approach to include bacterial cultures after the sorting of glycan^+^ cells to isolate consumers in the studied fecal sample. This allowed us to directly validate growth in vitro using the bacterial strains present in the samples and avoid the issue of inferring a strain based on a portion of the 16S rRNA gene. In a proof-of-principle study, we isolated 4 clones from 3 different phyla that could consume GMP and FOS as a sole carbon source in culture. The clones were identified by Sanger sequencing of the 16S rRNA gene as a *B. xylanisolvens* (Bacteroidetes) growing on GMP and *B. angulatum* (Actinobacteria), *B. uniformis* (Bacteroidetes), and *E. coli* (Proteobacteria) growing on FOS. While these consumers were known, more extensive culturomics with different and more specific growth media could yield unknown consumers^72^. In addition, the aerobic environment of the sorting step is likely detrimental to many strict anaerobes, and sorting under anoxic conditions will most likely improve the recovery of live bacteria labeled by fluorescent glycans.

Existing approaches to study bacterial glycan metabolism have typically focused on simplified complex communities, which can be hard to reproduce despite providing unparalleled insight^32,33^. Our approach complements such studies by using whole human fecal samples, which could also be patient-derived to capture different bacterial taxa. As our pipeline is functional and includes a cell sorting step, dead or damaged cells in the original samples are not labeled even if they encode the appropriate metabolic machinery. Thus, our method is complementary to genomic methods that do not discriminate between active, inactive, live, or damaged cells^34^. Expanding this approach to other glycans and samples can help further annotate PUL specificity and discover metabolic genes and pathways.

Due to its nonintrusive nature, human stool sampling is a powerful surrogate for the gut microbiota, as bacteria can remain metabolically active when rapidly placed in anoxic conditions^35,73,74^. However, it should be recognized that stool samples do not provide information on the location of specific gut bacteria along the digestive tract or across the depth of the mucus layer and that its composition represents shed bacteria^75^. Furthermore, stool samples vary in composition according to stool consistency, which reflects differences in transit time and potentially different niche sampling^76^. Nevertheless, this pipeline should be applicable to other types of gut microbiota, such as colonoscopy biopsies.

Here, we described the use of a functional pipeline that identifies bacteria that take up specific glycans in complex gut microbiota samples, thus expanding our functional understanding of glycan metabolism in the human gut microbiota. Applying this pipeline to other glycan structures and to other human populations with distinct diets, as well as individuals with gastrointestinal diseases, could help identify putative consumers of dietary glycans and prebiotic structures. Furthermore, combining this method with culturomics is useful to isolate bacterial consumers without knowledge of the specific genes involved, potentially leading to the discovery of novel CAZymes. The method could also be coupled to other genomic analyses, such as metagenomics and single-cell genomics, to characterize glycan metabolism in non-Bacteroidetes bacteria and discriminate between metabolism for growth vs metabolism without growth.

## Methods

### Bacterial strains

*Lactobacillus acidophilus (Moro) Hansen and Mocquot (ATCC® 4356™)* (*L.a*) and *Klebsiella oxytoca M5A1* (*K.o*), which were purchased from the American Type Culture Collection (Rockville, MD), were cultured at 37 °C in De Man, Rogosa, Sharpe (MRS) medium and Luria-Bertani (LB) medium, respectively.

### Synthesis

The detailed synthesis of the fluorescein-conjugated glycans is provided in the supporting information, along with the HPLC chromatograms.

### Human stool sample collection

Protocol A04-M27-15B was approved by the McGill Faculty of Medicine Institutional Review Board. We have received informed written consent from the participants for the use of human samples. Healthy participants with a body mass index between 18.5–30, no diagnosed gastrointestinal disease, no ongoing therapeutic treatment, and no antibiotic usage 3 months prior to the start of the study were eligible. Subject information was recorded at the time of sampling. The age of donors ranged from 21–40 years. The initial experiments were performed on one sample from a male donor. The metabolic labeling-FACSeq pipeline was performed on samples from 1 female and 2 males aged 30–40 years, and the BMI ranged from 19–27 kg/m^2^. Fresh fecal samples were collected and placed immediately in an anaerobic chamber, aliquoted, and stored at −80 °C until use.

### Metabolic labeling of cultured *Klebsiella oxytoca*

Cultures in minimum medium M9 (MM M9) of *K.o* supplemented with 0.1% β-cyclodextrin were collected during exponential phase growth via centrifugation, washed in MM M9, and suspended in 195 µL of MM M9. *K.o*. were treated with 500 nM CD-F probes or an equal volume of MM M9 cells as a control. The cells were incubated with shaking for 30 min at 37 °C in the dark. Cells were collected via centrifugation at 15,000 *×* *g* for 5 min and were washed 2 times with 1 mL of MM M9. Pellets were resuspended in 200 μL of MM M9, and fluorescence was measured by using a Spark 10 M Tecan plate reader at an excitation wavelength of 485 nm following emission at 535 nm. Fluorescence measurements were performed in triplicate, and the mean values and standard deviations are displayed in the figures.

### Metabolic labeling of cultured *Lactobacillus acidophilus*

Cultures in custom MRS (cMRS) (peptone (10 g/L), yeast extract (5 g/L), sodium acetate (5 g/L), ammonium citrate (92 g/L), potassium phosphate (2 g/L), magnesium sulfate (0.2 g/L), manganese sulfate (0.05 g/L), Tween 80 (1 ml\L)) of *L.a* supplemented with 0.1% inulin were collected during exponential phase growth via centrifugation, washed in cMRS and suspended in 195 µL of cMRS. *L.a* were treated with 500 nM of NYST-F probes or an equal volume of cMRS as a control. The cells were incubated with shaking for 30 min at 37 °C in the dark. Cells were collected via centrifugation at 15,000 *×* *g* for 5 min and washed 2 times with 1 mL of phosphate-buffered saline (PBS). Pellets were resuspended in 200 μL of PBS, and fluorescence was measured by using a Spark 10 M Tecan plate reader at an excitation wavelength of 485 nm following emission at 535 nm. Fluorescence measurements were performed in triplicate, and the mean values and standard deviations are displayed in the figures.

### Fluorescence microscopy

After carbohydrate labeling, *K.o*. or *L.a*. were fixed for 30 min with a solution of 4% paraformaldehyde, washed with a PBS solution and mixed at equal volumes with ProLong Gold antifade reagent (Invitrogen). Fixed bacteria were mounted on a slide and coverslip, and images were acquired with a LEICA SP8 confocal microscope (for *K.o*) and a Leica DM100 microscope (for *L.a*) and processed by Leica LAS X software.

### Flow cytometry analysis

Flow cytometry analysis was performed on a 5-laser LSR Fortessa 20-parameter analyzer. Cell sorting was performed on 3 lasers, 13 detector FACSAria-III or 4 lasers, and 18-detector FACSAria Fusion. Data were analyzed using BD FACSDiva or FlowJo software. To set the thresholds to specifically detect the cells labeled by the fluorescent probes, we used unlabeled cells as negative controls. The detection threshold for signals in the FITC channel was set at 10^3^ compared to the maximum signal generated by the negative control at 3 × 10^2^. The gating threshold may be adjusted at different levels to adjust the sensitivity of the assay depending on the probes. Fifty thousand or 100,000 events per sample were analyzed, and FITC fluorescence was measured using the FITC channel with excitation at 488 nm and emission at 535 nm. FSC and SSC (forward and side scatter, respectively) gating was performed to exclude doublets. A total of 1 to 3 × 10^6^ cells for each sample were sorted.

### Carbohydrate labeling

All labeling experiments were performed under anaerobic conditions (87% N_2_, 10% CO_2_, and 3% H_2_). A 0.1 g fecal sample was weighed and diluted to 1:10 g/mL in minimum medium (MM) (6.6 mM KH_2_PO_4_ (pH 7.2), 15 mM NaCl, 100 μM MgCl_2_, 175 μM CaCl_2_, 50 μM MnSO_4_, 5 mM (NH_4_)_2_SO_4_, 15 μM FeSO_4_, 24 µM NaHCO_3_, 1 g/L^−1^ L-cysteine, 1.9 μM hematin, 6 µM Hemin, and 200 ng/ml^−1^ vitamin B_12_), vortexed thoroughly and centrifuged for 3 min at 700 × *g*. The supernatant was saved, and the pellet was discarded. The supernatant was centrifuged for 5 min at 6500 × *g*, the supernatant was discarded, and the pellet was washed with MM. The pellet was resuspended in 200 µL of MM. Carbohydrate probes were added at a final concentration of 4.4 µM. Incubation was performed at 37 °C for one hour protected from light. The cells were then centrifuged for 5 min at 6500 × *g*, the supernatant was discarded, and the pellet was washed twice with reduced PBS (rPBS). The final pellet was resuspended in 500 µL of rPBS and kept at 4 °C protected from light. The bacterial suspension was then diluted in rPBS for flow cytometry analysis.

### Competition experiment with free cyclodextrin

Metabolic labeling was performed as above but with the addition of unlabeled β-cyclodextrin (218 µM, 436 µM, 872 µM, and 1.74 mM) with the CD-F probe. The fluorescence level was measured by flow cytometry.

### Heat inactivation

Heat pretreatment was performed after the preparation of the bacterial pellet from the fecal sample as described above. The incubation was for 10 min at 65 °C followed by cooling at 37 °C before metabolic labeling was performed as described above.

### Catabolic repression

An incubation in MM supplemented with 0.1% glucose for one hour was performed before metabolic labeling as described above.

### DNA extraction, 16S ribosomal RNA gene amplification, and MiSeq sequencing

DNA was extracted from all samples using the AllPrep PowerFecal DNA/RNA kit (Qiagen, Canada) following the manufacturer’s instructions. DNAs were quantified by the Qubit Fluorometric Quantification method (Invitrogen). The V4 region (based on *Escherichia coli*) of the 16S ribosomal RNA (rRNA) was targeted for amplification by PCR using the forward primer 515F GTGCCAGCMGCCGCGGTAA and the reverse primer 806R GGACTACHVGGGTWTCTAAT. The CS1 (ACACTGACGACATGGTTCTACA) and CS2 (TACGGTAGCAGAGACTTGGTCT) tags were used to add a barcode and Illumina adapters. Amplification was performed using Q5 High Fidelity DNA polymerase (New England BioLabs) with PCR cycles as follows: initial denaturation step of 98 °C for 30 s, before 23 cycles of 98 °C for 10 s, 58 °C for 15 s and 72 °C for 30 s, with a final extension at 72 °C for 2 min. The MiSeq platform was used for 2 × 250 bp paired-end sequencing of the resulting PCR products.

### Sequencing analysis

The sequencing data analysis was performed using the ANCHOR pipeline^48^. Briefly, the potential amplicons were assembled from high-quality reads. Assembled contigs of all lengths were conserved to control for unexpected amplicon length. Dereplicated sequences were evaluated, and sequences with a count higher than 9 were used as a confident basis for analysis (anchor threshold). These were processed and annotated against four major sequence repositories (curated NCBI bacterial and Archaea RefSeq database, NCBI nr/nt, SILVA, Ribosomal Database Project) with >99% identity and coverage thresholds. α- and β-diversity and ordination were analyzed using R scripts with the vegan library, and differential abundance analysis was performed using DESeq2^49^.

### Metagenomics data processing

Shotgun metagenomics data for three samples (MX73_1, MX73_2, and MX73_3) were preprocessed using MicrobiomeAnalystR, an R package for end-to-end data processing, statistical analysis, and visualization of microbiome sequencing data (https://github.com/xia-lab/MicrobiomeAnalystR)^77^. The MicrobiomeAnalystR metagenomic preprocessing pipeline integrates fastp^78^, BBMap (https://escholarship.org/uc/item/1h3515gn), and Kaiju^79^ to perform quality checks, host sequence decontamination, and taxonomic profiling, respectively. First, fastp was used for quality control, adapter trimming and quality filtering. Second, human reads were removed using BBMap by mapping the filtered reads against the hg38.fa reference genome. Finally, taxonomic classification of the remaining reads to the NCBI RefSeq Database was performed using Kaiju.

Mapping 16S rRNA sequences to metagenomic reads. BBduk (https://jgi.doe.gov/data-and-tools/bbtools/bb-tools-user-guide/bbduk-guide/) was used to map metagenomic reads (length of 100 bp) to 16S rRNA sequences (length of ~290 bp) of the 9 most important microbial species (as shown in Fig. 3). Mapping was performed on the forward and reverse reads for each sample independently. A hdist of 0 was set, and no mismatches were allowed.

### Growth curves

Isolates were cultured anaerobically (atmosphere: 87% N_2_, 10% CO_2_, 3% H_2_) at 37 °C overnight in brain heart infusion (BHI) media. The overnight cultures were then diluted at 1/250 in rPBS, and 10 µL of diluted bacteria was inoculated in 240 µL of minimal medium supplemented with 0.1% of the appropriate carbohydrate. The growth was assessed in a 96-well plate. The OD_600_ was measured in a plate reader (EPOCH, Biotech) under anaerobic conditions and was recorded every 5 min for 72 h.

*C. aerofaciens*, *B. wexlerae,* and *A. communis* required modified medium and conditions to measure carbohydrate consumption. *B. wexlerae* were grown overnight in trypticase soja (TS) medium, and growth curves were generated as described above. *C. aerofaciens* were grown overnight in Gam media, and the growth was tested in MM supplemented with yeast extract (20 mg/mL), beef extract (10 mg/mL) and 0.1% of the appropriate carbohydrate. *A. communis* was grown for 72 h in Columbia media supplemented with 1.9 μM hematin, 6 µM hemin, 15 μM FeSO_4_ and 200 ng/ml^−1^ vitamin B_12_, K_1_, and K_3_. To measure the growth of *A. communis* over 96 h, we used the Columbia media described above but depleted starch and glucose. Thereafter, sugar-depleted Columbia media was used with the appropriate carbohydrate at 0.1% to measure sugar metabolism.

The negative control consisted of 250 µL of MM supplemented with 0.1% carbohydrate. Three replicates were performed for each condition (*N* = 3), and the growth curves were generated in GraphPad Prism 9.

### DNA extraction and 16S rRNA sequencing of isolated bacteria

DNA was extracted from 1 mL of overnight bacterial culture. The One-4-All Genomic DNA Miniprep Kit (Biobasic) was used to extract the DNA according to the manufacturer’s instructions. The 16S rDNA gene was amplified by PCR, and Sanger sequencing was performed by Génome Québec.

### Stool DNA extraction and shotgun sequencing

Total bacterial DNA from stools was extracted using the AllPrep PowerFecal DNA/RNA kit (Qiagen, Canada) following the manufacturer’s instructions. DNAs were quantified by the Qubit Fluorometric Quantification method (Invitrogen). DNA was sent to Génome Québec for Illumina HiSeq 4000 PE 100 bp sequencing for shotgun metagenomics.

### Culturomics

Stool bacteria were labeled for one hour by either GMP-F or NYST-F probes as described above. GMP-F- or NYST-F-labeled bacteria were sorted on a 4-laser 18-detector FACSAria Fusion for 5 min. Sorted fluorescent bacteria were immediately transferred to an anaerobic chamber. GMP-F-labeled bacteria were cultured on BHI and TSA blood agar plates for 72 h at 37 °C. After incubation, individual clones were isolated on BHI or TSA blood plates and challenged for their growth ability on MM supplemented with 0.1% GMP. GMP consumer clones were identified by sequencing the 16S rRNA with the primers 27F AGAGTTTGATCCTGGCTCAG and 1492R GGTTACCTTGTTACGACTT.

After sorting, the NYST-F-labeled bacteria were suspended in 5 mL of ABB medium supplemented with 0.1% FOS (ABB-FOS) and incubated for 24 h at 37 °C. Following incubation, 100 µL aliquots of ABB-FOS culture were then transferred to 5 mL of MM supplemented with 0.1% FOS (MM-FOS) and incubated for 48 h at 37 °C. Diluted aliquots of positive cultures on MM-FOS were then spread on ABB-FOS, BHI or TSA blood plates and incubated for 48 h at 37 °C. Individual clones were then isolated on ABB-FOS, BHI and TSA blood plates and challenged for their growth ability on MM supplemented with 0.1% FOS. FOS consumer clones were identified by sequencing the 16 rRNA with the primers 27 F and 1492 R. Five milliliters of fresh ABB-FOS was added to the remaining ABB-FOS cultures and incubated for 24 h at 37 °C. Aliquots of this culture were incubated in 5 ml MM-FOS, and further clones were isolated as described above. A total of five passages were performed.

### Statistical analysis

No statistical method was used to predetermine sample size. No data were excluded from the analyses. All replicates are independent biological replicates with a minimum of *n* = 3. Statistical analysis was performed using GraphPad Prism 9.3.0. Statistical significance of labeling experiments of *K. oxytoca* and *L. acidophilus* compared to control (Fig. 1d) was determined by unpaired t-test with Bonferroni-Dunn’s multiple comparison test. All other labeling experiments (Figs. 1, 2, 4, and 5) were determined by one-way ANOVA with Bonferroni’s multiple comparison test comparing all columns to the labeled sample. The differential abundance analysis comparing the ESV abundance in the starting stool versus the glycan^+^ samples in Fig. 3 was determined by a DESeq2 analysis and is shown using the adjusted *P* value. The experiments were not randomized as all three stool samples were incubated with the three fluorescent glycans. All statistical tests performed are detailed in the corresponding figure captions.

### Reporting summary

Further information on research design is available in the Nature Portfolio Reporting Summary linked to this article.

## Supplementary information

Supplementary Information

Description of Additional Supplementary Files

Supplementary Data 1

Reporting Summary

## Data Availability

The 16S sequencing and metagenomic sequencing data generated in this study have been deposited in the National Center for Biotechnology Information (NCBI) database under Bioproject accession code PRJNA925842. The sequences for isolated bacteria reported in this paper have been deposited in the NCBI GenBank database under accession numbers: *B. xylanisolvens* CLD22001 (OP510057), *B. angulatum* CLD22003 (OP512543), *B. caccae* CLD22004 (OP512560), *B. uniformis* CLD22005 (OP514970), and *E. coli* CLD22006 (OP514723). The ANCHOR ESV sequences are available as a separate file (Supplementary Data 1). Source data and statistical details are provided as a Source data file. Source data are provided with this paper.

## Source data

Source Data
