# Supplementary material for: Identifying glycan consumers in human gut microbiota samples using metabolic labeling coupled with fluorescence-activated cell sorting
Source: Nat Commun. 2023 Feb 7;14:662. doi: 10.1038/s41467-023-36365-8 (PMC9905522; doi:10.1038/s41467-023-36365-8)
Supplement: Supplementary file 1 — Supplementary Information [file 41467_2023_36365_MOESM1_ESM.pdf]

Supplementary Information

Identifying Glycan Consumers in Human Gut Microbiota Samples using Metabolic Labeling Coupled with Fluorescence-Activated Cell Sorting

Lharbi Dridi[1], Fernando Altamura[1], Emmanuel Gonzalez[2,3,4], Olivia Lui[1], Ryszard Kubinski[1], Reilly Pidgeon[1], Adrian Montagut[1], Jasmine Chong[5], Jianguo Xia[5], Corinne F. Maurice[6,7]*, Bastien Castagner[1]*
[1]Department of Pharmacology & Therapeutics, McGill University, 3655 Prom. Sir-William-Osler, Montreal, Quebec, H3G 1Y6, Canada.
[2]Canadian Centre for Computational Genomics, McGill Genome Center, 740, Dr. Penfield Avenue, Montreal, Quebec, H3A 0G1, Canada.
[3]Department of Human Genetics, McGill University, 3640 University, Montreal, Quebec, H3A 0C7, Canada
[4]Gerald Bronfman Department of Oncology, McGill University, 5100 Boulevard de Maisonneuve West, Montreal, Quebec, H4A 3T2, Canada
[5]Institute of Parasitology, McGill University, 21111 Lakeshore Rd, Ste-Anne-de-Bellevue, Quebec, H9X 3V9, Canada
[6]McGill Centre for Microbiome Research, McGill University, Montreal, Quebec, Canada
[7]Department of Microbiology & Immunology, McGill University, 3773 University Street, Montreal, Quebec, H3A 2B4, Canada.

*Correspondence: bastien.castagner@mcgill.ca and corinne.maurice@mcgill.ca

Table of contents

Supplementary Table 1 2
Synthesis, purification, and characterization of fluorescent glycans 4
Synthesis of CD-F (1) 5
Synthesis of NYST-F (2) 5
Synthesis of GMP-F (3) 6
Synthesis of Glucose-F (4) 6
Synthesis of Maltose-F (5) 6
Synthesis of ARAB-F (6) 7
Synthesis of FOS-F (7) 7
Synthesis of MANNO-F (8) 8
Synthesis of XYLO-F (9) 8
Synthesis of ARABXYLO-F (10) 9
Supplementary Figures 10
References 18
Chromatograms of fluorescent glycans 19

Supplementary Table 1

Reagent or Resource	Source	Identifier
Lactobacillus acidophilus (Moro) Hansen and Mocquot (ATCC® 4356™)	ATCC	Cat#ATCC 4356
Klebsiella oxytoca M5A1	ATCC	Cat#ATCC BAA-1236
Bacteroides vulgatus ATCC 8482	ATCC	Cat#ATCC 8482
Alistipes communis	DSMZ	DSM 108979
Blautia wexlerae	DSMZ	DSM 19850
Collinsella aerofaciens	DSMZ	DSM 3979
Human Fecal Sample	McGill University	EY42
Human Fecal Sample	McGill University	MX73
Human Fecal Sample	McGill University	PY31
Human Fecal Sample	McGill University	YM54
MRS	Sigma-Aldrich	Cat#69966
M9	Bioshop	Cat#MIS409
LB BROTH (MILLER)	Bioshop	Cat#LBL407
ANAEROBE BASAL BROTH	Oxoid	CM0957
TSA with Sheep Blood	Thermo Fisher Scientific	RE01200
BHI	BD	237500
Sodium chloride	Bioshop	Cat#SOD002
Potassium phosphate monobasic	Bioshop	Cat#PPM302
Magnesium chloride	Bioshop	Cat#MAG510
Calcium chloride	Fischer	Cat#BP510
Manganese sulfate	Bioshop	Cat#MAN111
Ammonium sulfite	Bioshop	Cat#AMP302
Sodium Bicarbonate	Bioshop	Cat#SOB308
Ferrous Sulfate Heptahydrate	Bioshop	Cat#FER005
Hematin	Sigma-Aldrich	Cat#H3281
Hemin	Sigma-Aldrich	Cat#H9039
Vitamin B12	Bioshop	Cat#VIT271
Peptone	Bioshop	Cat#PEP403
Yeast extract	Bioshop	Cat#YEX555
Beef extract	Bioshop	Cat#BEF222
Sodium acetate	Bioshop	Cat#SAA305
Ammonium citrate	Sigma-Aldrich	Cat#25102
Potassium phosphate	Bioshop	Cat#PPD303
Magnesium sulphate	Bioshop	Cat#MAG511
Manganese sulphate	Bioshop	Cat#MAN111
Tween 80	Bioshop	Cat#TWN507

PBS 10X, PH 7.4	Wisent	Cat#311-012-CL
UltraPure™ DNase/RNase-Free Distilled Water	Thermo Fisher Scientific	Cat#10977015
Inulin from chicory	Sigma-Aldrich	Cat#I2255
β-Cyclodextrin	Sigma-Aldrich	Cat#C4767
prolong gold antifade reagent	Thermo Fisher Scientific	Cat#P10144
Formaldehyde 37%	Fisher	Cat#BP531
One-4-All Genomic DNA Minipreps kit	Biobasic	BS88503
AllPrep PowerFecal DNA/RNA Kit	Qiagen	Cat#80244
Qubit dsDNA HS assay Kit	Thermo Fisher Scientific	Cat#Q32851
Taq DNA Polymerase with ThermoPol® Buffer	NEB	M0267S
Sodium hydride (60% in mineral oil)	Millipore Sigma Canada	CAS No: 7646-69-7
N,*N*-Dimethylformamide	Fischer Chemical	CAS No: 68-12-1
p-toluenesulfonyl chloride	Millipore Sigma Canada	CAS No: 98-59-9
Ethyl-Acetate	Fischer Chemical	E145
Ethyl alcohol anhydrous	Greenfield	P016EAAN
Sulfuric acid	Fischer Chemical	A300
Molecular Sieves, Grade 514	Fischer Scientific	CAS No: 1344-00-9
Fluorescein-NHS (5/6-carboxyfluorescein succinimidyl ester), mixed isomer	Thermo-Scientific	CAS No: 46410
Nystose	Millipore Sigma Canada	CAS No: 13133-07-8
1,5-α-L-Arabinotetraose	Megazyme	CAS No: 190852-24-5
Xylotetraose	Megazyme	CAS No: 22416-58-6
Mannotetraose	Megazyme	CAS No: 51327-76-5
3^3-α-L-arabinofuranosyl-xylotetraose	Megazyme	CAS No: 84666-93-3
6^3,6^4-α-D-galactosyl-mannopentaose	Megazyme	CAS No: 86781-72-8
D-Glucose Monohydrate	BioShop Canada	CAS No: 14431-43-7
D-(+)-Maltose monohydrate	Millipore Sigma Canada	CAS No: 6363-53-7
Fructooligosaccharides from chicory	Millipore Sigma Canada	F8052-50G
Levan	Megazyme	P-LEVAN
PD-10 Desalting Columns containing Sephadex G-25	GE Healthcare Life Sciences	
Prism v9	GraphPad Software	http://www.graphpad.com/scientific-software/prism/

FlowJo	FlowJo	N/A
MicrobiomeAnalystR	Xia-Lab	https://github.com/xia-lab/MicrobiomeAnalystR).
BD FACSDiva	BD	N/A
LSR Fortessa	BD	N/A
FACSAria-III	BD	N/A
FACSAria Fusion	BD	N/A
Leica DM100	LEICA	N/A
Leica LAS X	LEICA	N/A
Epoch 2	Biotek	N/A
Spark 10M	Tecan	N/A
Vinyl Anaerobic Chambers	COY	N/A
LEICA SP8 confocal	LEICA	N/A
1260 Infinity LC system connected to a 6120 Quadrupole LC/MS mass spectrometer	Agilent	N/A

Synthesis, purification, and characterization of fluorescent glycans

All chemicals were reagent grade and used as supplied unless stated otherwise. Solvents for reactions (*N*,*N*-dimethylformamide, acetone, methanol, ethanol, ethyl acetate) were purchased of analytical grade from commercial suppliers and used without further purification unless stated otherwise. Solvents for extractions and chromatography were technical grade. Ultrapure water was prepared by a MilliQ system from Millipore (Millipore Sigma Canada). Analytical thin layer chromatography (TLC) was performed on pre-coated Merck silica gel 60 F254 plates and visualized with UV light or cerium molybdate stain. Flash column chromatography was carried out on Sigma-Aldrich silica gel 60Å (230-400 mesh). For reverse phase column chromatography, a 1260 Infinity LC system connected to a 6120 Quadrupole LC/MS mass spectrometer from Agilent Technologies was used. For analytical runs, an X-Select CSH C18 5 µm x 3 µm x 150 mm column was used. For preparatory runs, an X-select CSH C18 OBD 130 Å 5 µm x 10 mm x 150 mm column was used. In both analysis and preparatory runs, an increasing 13 min (0-100%) acetonitrile gradient was used at a flow rate of 0.425mL/min for analytical and 4.72mL/min for preparatory run. Retention time for the products in HPLC was determined using the 280nm spectrum of the analytical runs. Size exclusion column chromatography was performed on PD-10 Desalting Columns containing Sephadex G-25 (GE Healthcare

Life Sciences, Buckinghamshire, UK). Lyophilizations were performed on a Christ Freeze Dryer Alpha 2-4 LSC (Montreal Biotech, Canada). ^{1}H NMR spectra were recorded on a Bruker AV 400 MHz spectrometer NMR data are reported as follows: chemical shifts (δ, ppm, relative to residual solvent peaks), integration, multiplicity (s = singlet, br = broad, d = doublet, t = triplet, q = quartet, m = multiplet), coupling constant (J, Hz). MALDI-TOF spectra were captured on UltrafleXtreme MALDI TOF/TOF (Brucker). Purity assessment of was performed via peak integration of the 280nm spectrum from reverse phase LC-MS.

Synthesis of CD-F (1)

β-cyclodextrin was tosylated and mono-functionalized with ethylene diamine as reported previously.(Yang et al., 2008) 10mg of ethylene diamine-β-CD were dissolved in 200µL of 70 mM HEPES buffer, and 15µL of a 1N HCl solution were added to reach pH 7.0. Two mg of 5(6)-carboxyfluorescein diacetate *N*-succinimidyl ester (Fl-NHS) dissolved in 0.1 mL of *N*,*N*-dimethylformamide (DMF) were added to the solution, and the resulting mixture was shaken overnight at room temperature. The reaction crude was analyzed using reverse-phase analytical liquid chromatography-mass spectrometry (LC-MS), and the elution peak corresponding to CD-Fl was fractionated via preparative high-pressure liquid chromatography (HPLC). The purity of the fraction was finally assessed with reverse-phase analytical LC-MS [m/z (ESI) (M+2H) $C_{65}H_{88}N_2O_{40}$ calculated 768.24, found 768.3 retention time: 11-12min].

Synthesis of NYST-F (2)

Five mg of nystose were suspended in 200µL of DMF and shaken at maximum speed for 30 minutes at 50°C. After cooling, 2µL of triethylamine (NEt_3), 4-(dimethylamino)pyridine (DMAP) (cat.) and 6-9 beads of 4Å molecular sieves were added, and the resulting mixture was left to shake overnight at room temperature, and at maximal speed. On the next day, 1.5mg of Fl-NHS was added, and the mix was shaken again for 2 extra hours. The reaction mixture was diluted with water to reach a volume of 1.5mL and passed through a PD-10 desalting column (GE Healthcare). After eluting with water, 10 coloured fractions were collected, lyophilized, and analysed using reverse-phase analytical LC-MS. Those containing NYST-F were pooled together, and the elution peak

corresponding to the mono-functionalized product was fractionated via preparative HPLC. The final fraction was analyzed using reverse-phase analytical LC-MS and MALDI-TOF [m/z (ESI) (M+H) $C_{45}H_{53}O_{27}$ calculated 1025.28, found 1025.33 retention time: 8-9.5min].

Synthesis of GMP-F (3)

Four mg of 6^3,6^4-α-D-galactosyl-β(1,4)-D-mannopentaose were suspended in 200μL of DMF and shaken at maximal speed for 30 minutes at 50°C. After cooling, 2μL of NEt_3, DMAP (cat.) and 6-9 beads of 4Å molecular sieves were added, and the resulting mixture was left to shake overnight at room temperature, and at maximal speed. On the next day, 3 equivalents of Fl-NHS were added to the mix that was later shaken for an additional 24 hours. On the next day, the crude reaction was eluted trough a silica column (Sigma Aldrich) with a gradient of EtOAc:EtOH (1:1) with increasing concentration of water. The fractions were analysed using reverse-phase analytical LC-MS, and those containing the mono-functionalized sugar were pooled and fractionated via preparative HPLC. The purity of the fraction was finally assessed with reverse-phase analytical LC-MS and MALDI-TOF [m/z (M+K) $C_{63}H_{82}O_{42}K$ calculated 1549.39, found 1549.64, retention time: 7.5-8.5 min]

Synthesis of Glucose-F (4)

D-Glucose (4mg, 1eq.) was suspended in 200μL of DMF and shaken at maximum speed for 30 minutes at 50°C. After cooling, 2μL of Et_3N, DMAP (cat.) and 6-9 beads of 4 Å molecular sieves were added. The resulting mixture was left to shake overnight at room temperature, and at maximal speed. On the next day, the sieves were removed and 5.3 mg Fluorescein-NHS (0.5eq) was added. The mix was shaken for 24 hours. The crude reaction was then analyzed via LC-MS and then fractionated via preparative HPLC. The purity of the fraction was finally assessed with reverse-phase analytical LC-MS [m/z (ESI) (M+H) $C_{27}H_{23}O_{12}$ calculated 539.12, found 539.0, retention time: 10.5-11.25 min].

Synthesis of Maltose-F (5)

D-(+)-Maltose Monohydrate (4mg, 1eq.) was suspended in 200μL of DMF and shaken at maximum speed for 30 minutes at 50°C. After cooling, 2μL of Et_3N, DMAP (cat.) and 6-9 beads of 4 Å molecular sieves were added. The resulting mixture was left to shake

overnight at room temperature, and at maximal speed. On the next day, the sieves were removed and 4.4 mg Fluorescein-NHS (0.5eq) was added. The mixture was shaken for 24 hours. The crude reaction was then analyzed using LC-MS and fractionated via preparative HPLC. The purity of the fraction was finally assessed with reverse-phase analytical LC-MS [m/z (ESI) (M+H) $C_{33}H_{33}O_{17}$ calculated 701.17, found 701.2, retention time: 10-11min].

Synthesis of ARAB-F (6)

Four mg of α(1-5)-L-arabinotetraose were suspended in 200µL of DMF and shaken at maximum speed for 30 minutes at 50°C. After cooling, 6-9 beads of 4Å molecular sieves and 3 equivalents of sodium hydride (NaH) were added, and, after a quick mix, 3 equivalents of Fl-NHS. The mixture was shaken overnight at room temperature, and at maximal speed. On the next day, the reaction was acidified with Dowex 50WX8 resin for a few hours on a rocking plate. The mix was eluted trough a silica column (Sigma Aldrich) with a gradient of EtOAc:EtOH (1:1) with increasing concentration of water. The fractions were analysed using reverse-phase analytical LC-MS, and those containing the mono-functionalized sugar were pooled and fractionated via preparative HPLC. The purity of the fraction was finally assessed with reverse-phase analytical LC-MS [m/z (ESI) (M+H) $C_{41}H_{45}O_{23}$ calculated 905.23, found 905.3, retention time: 9-10 min].

Synthesis of FOS-F (7)

Two mg of fructo-oligosaccharide was suspended in 200µL of DMF and shaken at maximum speed for 30 minutes at 50℃. After cooling to room temperature, 6-9 beads of 4 Å molecular sieves were added. The resulting mixture was left to shake overnight at room temperature, and at maximal speed. On the next day, the sieves were removed and 0.5 mg of NaH (60% in mineral oil) and 5.7 mg Fluorescein-NHS was added. The mixture was shaken for an additional 24 hours. The crude reaction was then acidified using 100 mg of DMF-washed Dowex 50WX8 resin for 6 hours, then fractionated into 1mL fractions using a 1cm x 42 cm Sephadex LH20 column and 50/50 water/acetonitrile as eluent. Fractions containing FOS were combined and absence of free fluorescein was confirmed via LC-MS. Functionalized sugar presence was confirmed with MALDI-TOF. Of the FOS

polymers visible on the MALDI spectrum, many FOS polymers appear to be functionalized with the Fluorescein group. Among these, the functionalized FOS_{10}-Fl appeared in different ionization states (Na^+ or K^+) FOS_{10}-Fl [(M+Na) $C_{81}H_{112}O_{57}Na^+$, calculated: 2020.08 found: 2020.981], FOS_{10}-Fl [(M+K) $C_{81}H_{112}O_{57}K^+$, calculated: 2036.08 found: 2035.951].

Synthesis of MANNO-F (8)

Four mg of β(1-4)mannotetraose were suspended in 200µL of DMF and shaken at maximum speed for 30 minutes at 50°C. After cooling, 6-9 beads of 4Å molecular sieves and 3 equivalents of sodium hydride (NaH) were added, and, after a quick mix, 3 equivalents of Fl-NHS. The mixture was shaken overnight at room temperature, and at maximal speed. On the next day, the reaction was acidified with Dowex 50WX8 resin for a few hours on a rocking plate. The mix was eluted trough a silica column (Sigma Aldrich) with a gradient of EtOAc:EtOH (1:1) with increasing concentration of water. The fractions were analysed using reverse-phase analytical LC-MS, and those containing the mono-functionalized sugar were pooled and fractionated via preparative HPLC. The purity of the fraction was finally assessed with reverse-phase analytical LC-MS Fl [m/z (ESI) (M+H) $C_{45}H_{53}O_{27}$ calculated 1025.28, found 1025.3, retention time: 8-9.25min].

Synthesis of XYLO-F (9)

Four mg of β(1-4)xylotetraose were suspended in 200µL of DMF and shaken at maximum speed for 30 minutes at 50°C. After cooling, 6-9 beads of 4Å molecular sieves and 3 equivalents of sodium hydride (NaH) were added, and, after a quick mix, 3 equivalents of Fl-NHS. The mixture was shaken overnight at room temperature, and at maximal speed. On the next day, the reaction was desalted and acidified with Dowex 50WX8 resin for a few hours on a rocking plate. The mix was eluted trough a silica column (Sigma Aldrich) with a gradient of EtOAc:EtOH (1:1) with increasing concentration of water. The fractions were analysed using reverse-phase analytical LC-MS, and those containing the mono-functionalized sugar were pooled and fractionated via preparative HPLC. The purity of the fraction was finally assessed with reverse-phase analytical LC-MS. [m/z (ESI) (M+H) $C_{41}H_{45}O_{23}$ calculated 905.23, found 905.2, retention time: 9.25-8.75min].

Synthesis of ARABXYLO-F (10)

Four mg of 3^3-α-L-arabinofuranosyl-β(1-4)xylotetraose were suspended in 200µL of DMF and shaken at maximal speed for 30 minutes at 50°C. After cooling, 2µL of NEt_3, DMAP (cat.) and 6-9 beads of 4Å molecular sieves were added, and the resulting mixture was left to shake overnight at room temperature, and at maximal speed. On the next day, 3 equivalents of Fl-NHS were added to the mix that was shaken for an additional 24 hours. On the next day, the crude reaction was eluted trough a silica column (Sigma Aldrich) with a gradient of EtOAc:EtOH (1:1) with increasing concentration of water. The fractions were analysed using reverse-phase analytical LC-MS, and those containing the mono-functionalized sugar were pooled and fractionated via preparative HPLC. The purity of the fraction was finally assessed with reverse-phase analytical LC-MS [m/z (ESI) (M+H) $C_{46}H_{53}O_{27}$ calculated 1037.28, found 1037.3, retention time: 8-9min].

Supplementary Figures

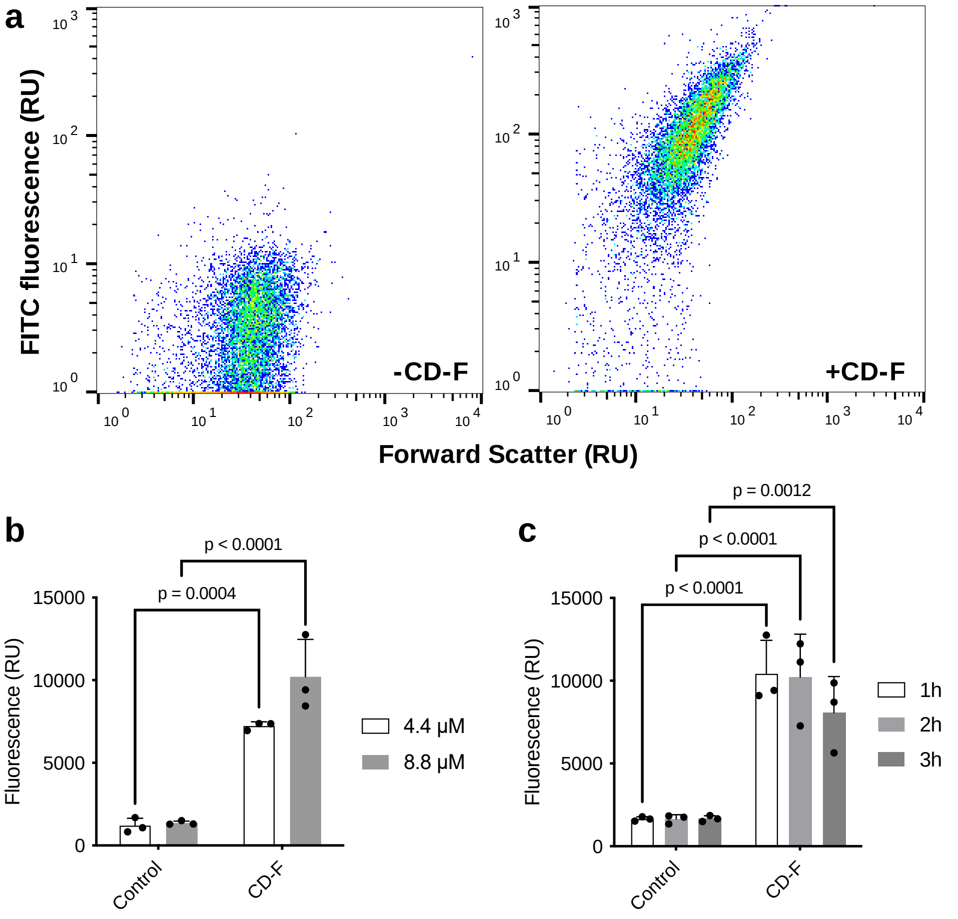

Figure S1. Characterization of CFD-F labeling on *K. oxytoca* and bacteria from stool. (**a**) Metabolic labeling of *K. oxytoca* with CD-F. Flow-cytometry pseudocolor plots of *K. oxytoca* grown in MM supplemented with non-fluorescent (left) and fluorescent (right) β-cyclodextrin for 30 min. Fluorescent quantification of bacteria isolated from stool samples after incubation with (**b**) 4.4 and 8.8 µM of CD-F for 1h in minimum media or (**c**) 1-3h with 4.4 µM of CD-F. Data are mean ± SD (n=3). Statistical significance compared to the control condition by two-way ANOVA with Šídák's multiple comparison test. Source data and statistical details are provided as a Source Data file.

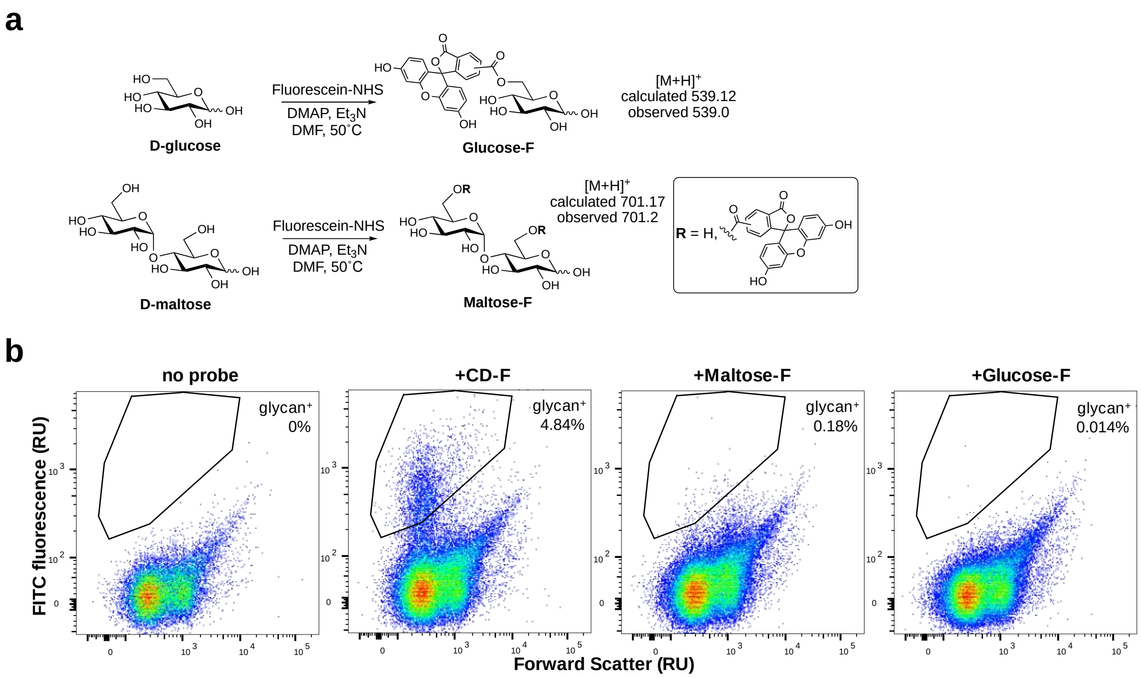

Figure S2. (**a**) Synthesis of glucose-F (**4**) and maltose-F (**5**). (**b**) Flow cytometry pseudocolor plots of stool bacteria incubated with CD-F, maltose-F or glucose-F.

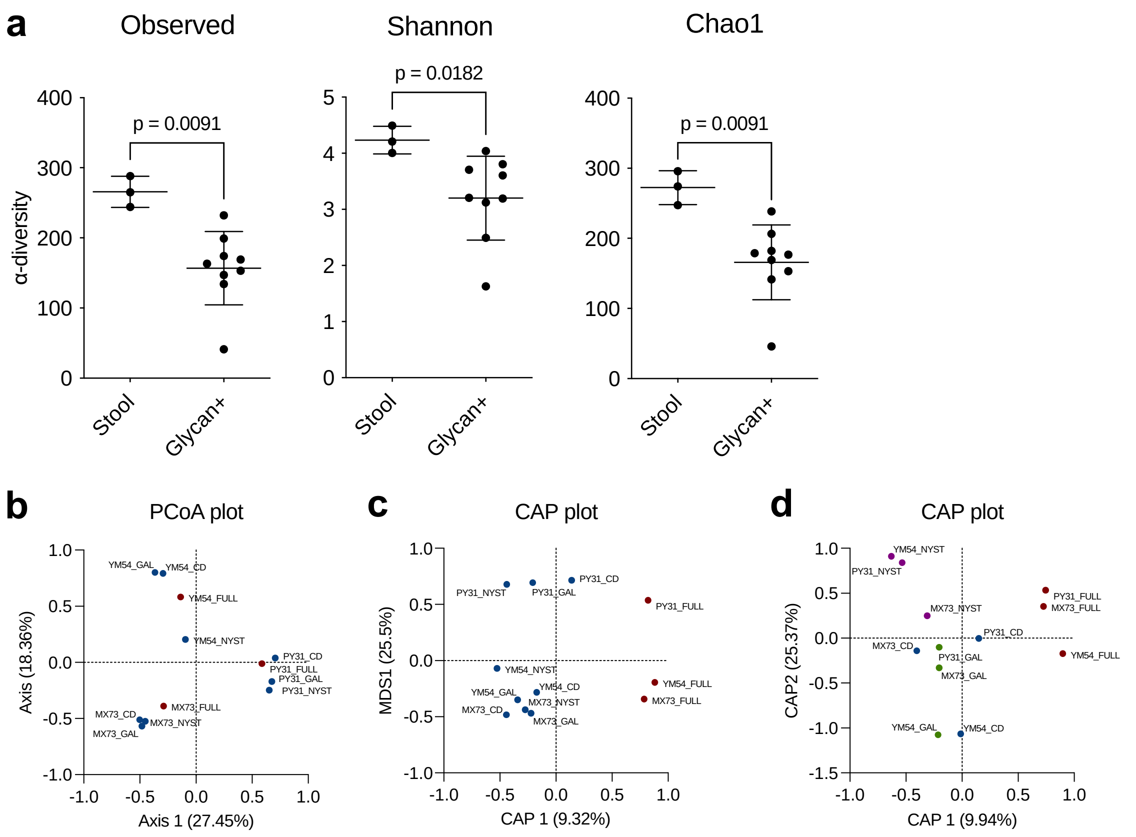

Figure S3. **α- and β-diversity of the initial stool samples and glycan⁺ sorted cells.** (**a**) α-diversity between initial stool samples (Stool) and glycan⁺ according to Observed, Chao1, and Shannon Indices. Data are shown as means ± SD. Statistical significance by Mann-Whitney test. (**b**) Principal coordinate analysis (PCoA) plot with Bray-Curtis dissimilarity of the initial stool samples (FULL) versus the glycan⁺ population in three different volunteers (MX73, YM54, and PY31). (**c-d**) Constrained analysis of principle coordinates (CAP) ordination method was used to visualize differences between populations in the initial stool samples versus glycan⁺ (**c**) or versus CD-F⁺, GAL-F⁺, and NYST-F⁺ (**d**). Source data and statistical details are provided as a Source Data file.

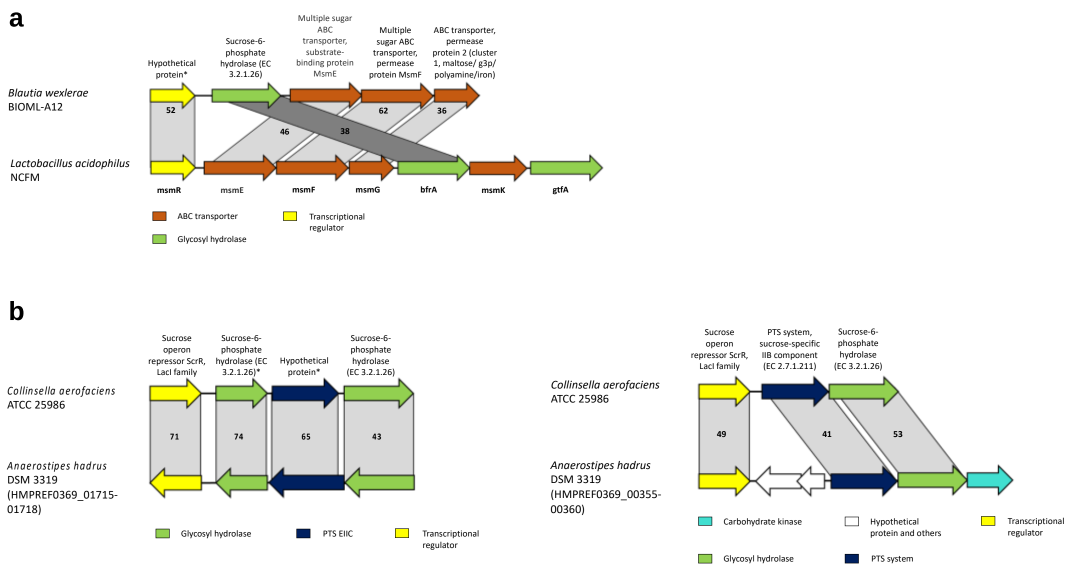

Figure S4. Comparisons of the homologous PULs. (**a**) Putative PUL in *B. wexlerae* BIOML-A12 and characterized PUL in *L. acidophilus* NCFM for FOS metabolism. Function of the proteins annotated in PATRIC 3.6.7 is indicated above the arrows for *B. wexlerae* BIOML-A12. Hypothetical protein* denotes a gene product that is annotated as "LacI family DNA-binding transcriptional regulator" in NCBI Protein. (**b**) Putative PULs in *C. aerofaciens* ATCC 25986 and characterized PULs in *A. hadrus* DSM 3319 for FOS metabolism. Function of the proteins annotated in PATRIC 3.6.7 is indicated above each gene. Sucrose-6-phosphate hydrolase (EC 3.2.1.26)* denotes a gene product that is annotated as "family 43 glycosylhydrolase" in NCBI Protein. Hypothetical protein* denotes a gene product that is annotated as "PTS transporter subunit EIIC" in NCBI Protein. RefSeq locus tag of *A. hadrus* DSM 3319 is indicated in brackets. Characterized genes annotated in PATRIC 3.6.7 are in bold under the arrows. Percent of amino acid sequence similarity is indicated within the shaded areas between corresponding proteins and was determined using EMBOSS Needle pairwise alignment (Needleman and Wunsch, 1970) with a BLOSUM62 matrix, gap open penalty = 10, and gap extend penalty = 1.

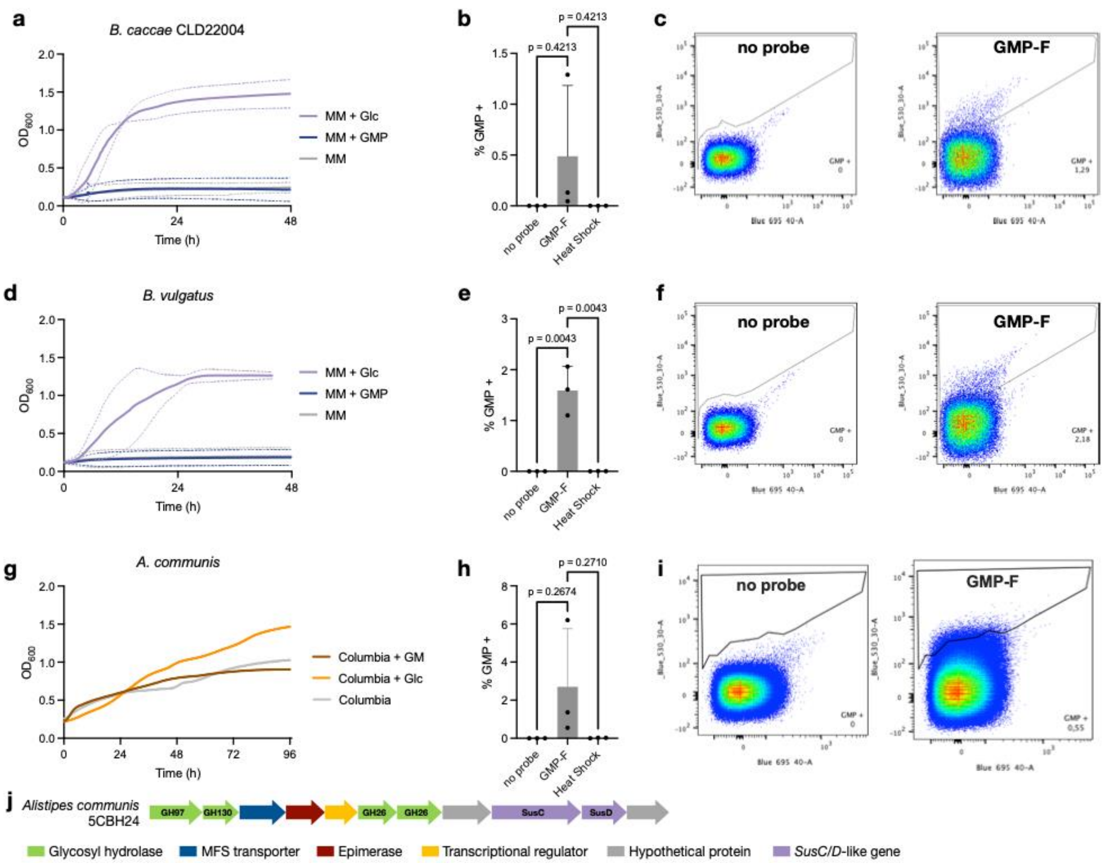

Figure S5. *B. caccae*, *B. vulgatus* and *A. communis* are not consumers of GMP. Bacterial growth of *B. caccae* CLD22004 (**a**), *B. vulgatus* (**d**) and *A. communis* (**g**) in MM with or without GMP. The viability of bacteria was evaluated by growth in MM supplemented with glucose. Uptake level of GMP-F, measured by flow cytometry, for *B. caccae* CLD22004 (**b**), *B. vulgatus* (**e**) and *A. communis* (**h**). Measurements were also conducted after a heat shock pretreatment of the bacteria to measure the specificity. Flow cytometer pseudocolor plot illustrating the GMP-F uptake by *B. caccae* CLD22004 (**c**), *B. vulgatus* (**f**) and *A. communis* (**i**). All measurements were done in triplicate, mean ± SD are displayed. Statistical significance compared to the probe condition by one-way ANOVA with Bonferroni's multiple comparison test. **j**) Identification of putative genes involved in the metabolism of GMP in *A. communis*. BLAST alignments were performed on complete genome available on NCBI database with known glycosyl hydrolase involved in the metabolism of GMP. Source data and statistical details are provided as a Source Data file.

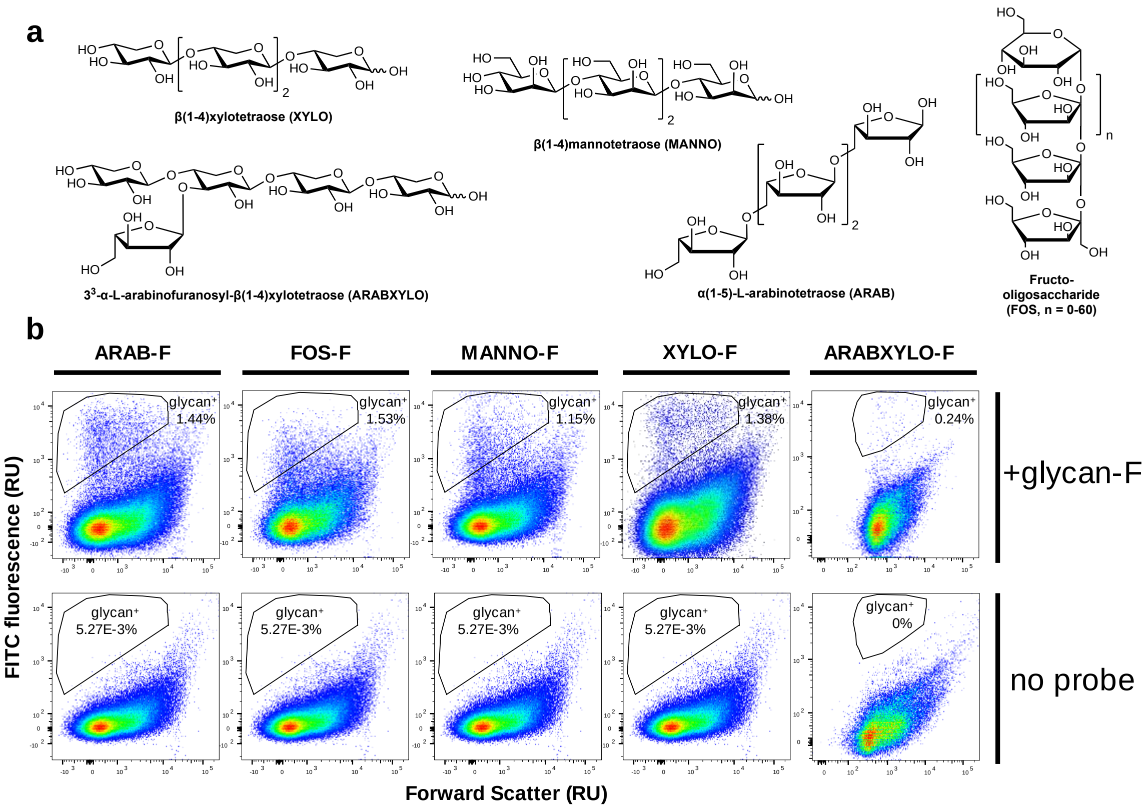

Figure S6. Structures and labeling with of a collection of glycans probes. (**a**) Structures of glycans used for the synthesis. (**b**) Representative flow cytometric pseudocolor plots of metabolic labeling of stool samples by ARA-F (**6**), FOS-F (**7**), MANNO-F (**8**), XYLO-F (**9**), and ARABXYLO-F (**10**). Bacteria isolated from stools were incubated with the different fluorescent glycan probes (at 4.4 μM) in MM under anaerobic conditions for 1h at 37°C.

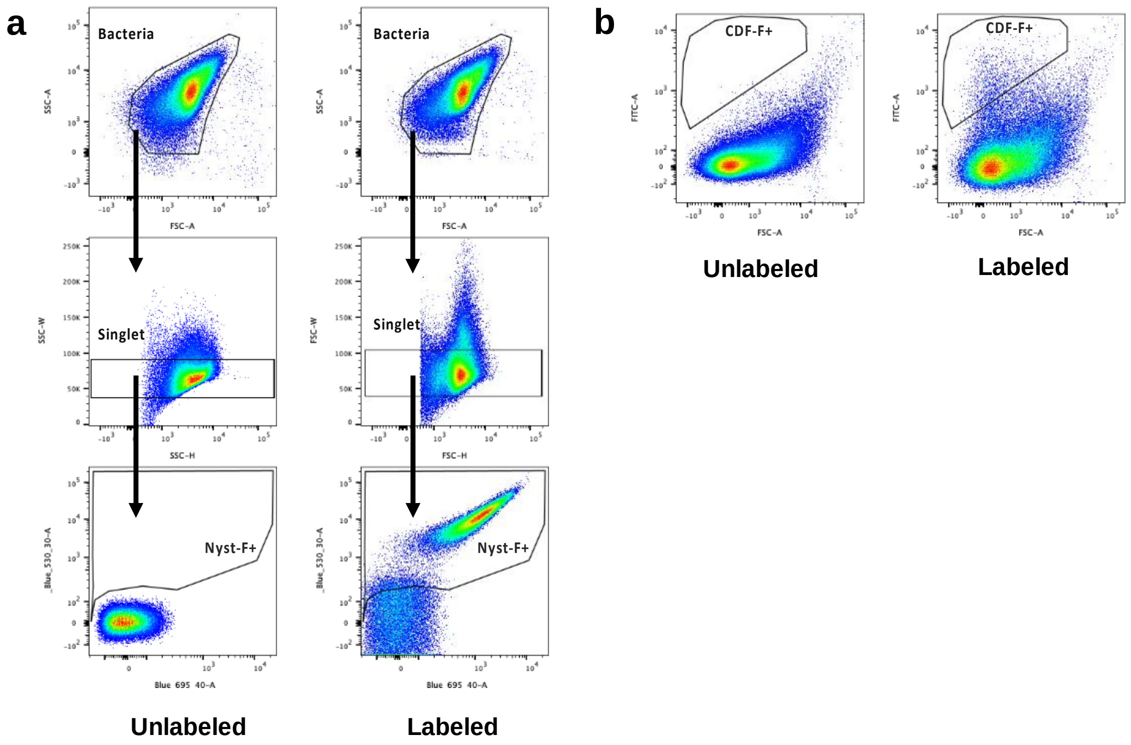

Figure S7. Gating strategy for flow cytometry. (**a**) Flow cytometry gating strategy for fluorescent bacteria isolates in figure 4; 5 and S5. (**b**) Flow cytometry gating strategy to identify the fluorescent *Klebsiella oxytoca* and bacteria isolated from stool in figure 2; S1; S2, and S6. Background fluorescence from unstained cells were used as negative controls to define positivity in Blue_530_30 or FITC channel.

Supplementary Table 2. Mapping ESVs sequences to metagenomic reads in MX73 stool sample.

ESVs	MX73_1_R1	MX73_1_R2	MX73_2_R1	MX73_2_R2	MX73_3_R1	MX73_3_R2
Alistipes_communis_1	80	68	29	19	21	18
Bacteroides_caccae_1	87	77	68	46	58	55
Bacteroides_uniformis_1	145	98	54	59	48	50
Bacteroides_vulgatus_1	1279	1192	806	766	1427	1279
Blautia_wexlerae_1	422	374	561	472	403	357
Clostridiales_MG_19	179	167	266	186	162	126
Collinsella_aerofaciens_1	254	214	504	489	403	350
Lachnospiraceae_MG_17	362	323	561	474	397	348
Parabacteroides_distasonis_2	9	7	3	2	5	3

Supplementary Table 3. Genome accession.

ESVs	genome match	% cov.	% ID	accession
Alistipes_communis_1	Alistipes communis 5CBH24 DNA, complete genome	100	100	AP019735.1
Bacteroides_caccae_1	Bacteroides caccae strain ATCC 43185 chromosome, complete genome	100	100	CP022412.2
Bacteroides_vulgatus_1	Bacteroides vulgatus ATCC 8482, complete genome	100	100	CP000139.1
Blautia_wexlerae_1	Blautia wexlerae strain BIOML-A12 scaffold109_size1998, whole genome shotgun sequence	100	100	GCF_009881435.1
Collinsella_aerofaciens_1	Collinsella aerofaciens ATCC 25986 strain JCM 10188 chromosome, complete genome	100	100	CP048433.1
Parabacteroides_distasonis_2	Parabacteroides distasonis CL03T12C09 chromosome, complete genome	100	100	CP072254.1
Prevotella_copri_1	Prevotella copri strain YF2 chromosome	100	100	CP042464.1
Prevotella_copri_2	no full genome it seems, YF2	100	99.66	CP042464.1

References

Needleman, S.B., and Wunsch, C.D. (1970). A general method applicable to the search for similarities in the amino acid sequence of two proteins. J Mol Biol *48*, 443-453.

Yang, M., Chu, L.Y., Wang, H.D., Xie, R., Song, H., and Niu, C.H. (2008). A thermoresponsive membrane for chiral resolution. Adv Funct Mater *18*, 652-663.

Chromatograms of fluorescent glycans

CD-F

A

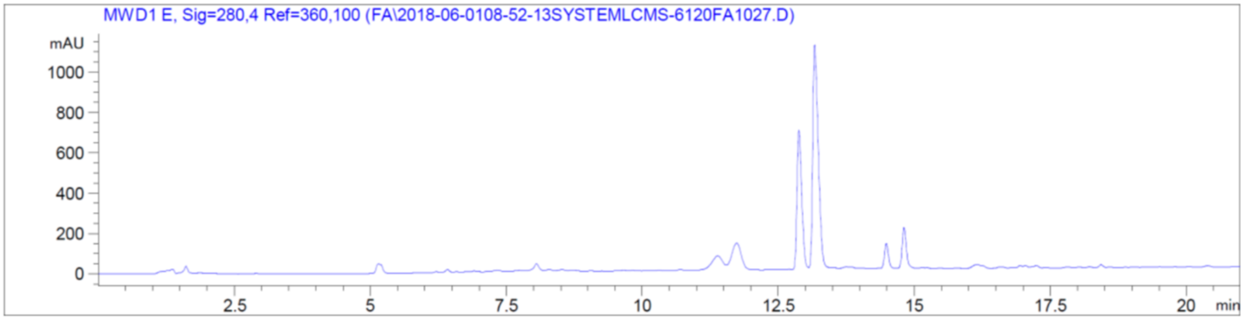

B

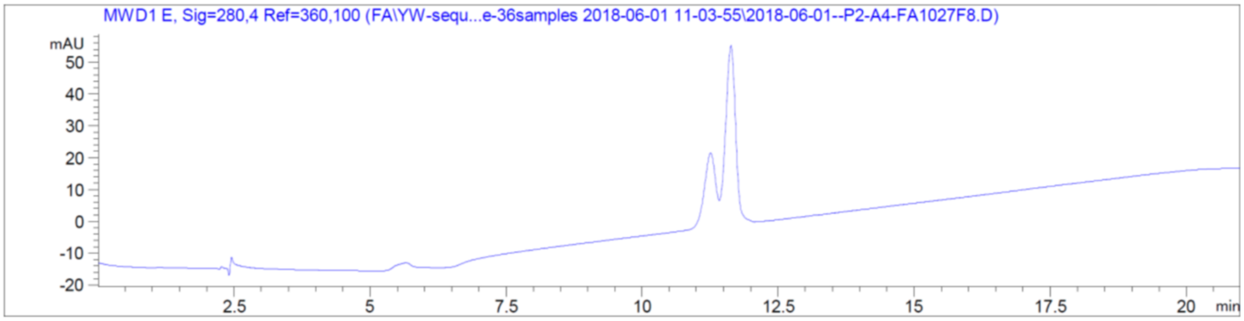

C

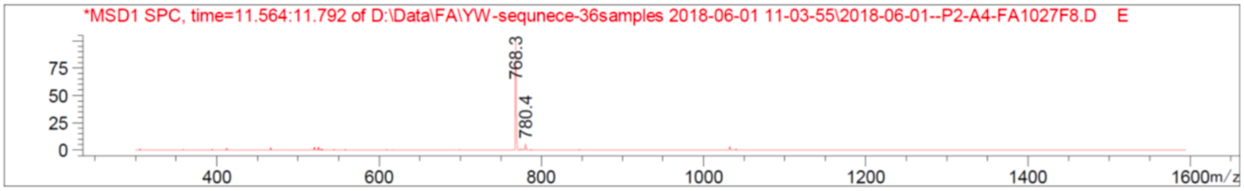

Spectra from the synthesis of CD-F. A) HPLC spectrum of crude CD-F. **B)** HPLC spectrum of purified CD-F. **C)** LCMS spectrum of purified CD-F [m/z (ESI) (M+2H) $C_{65}H_{88}N_2O_{40}$ calculated 768.24, found 768.3]

NYST-F

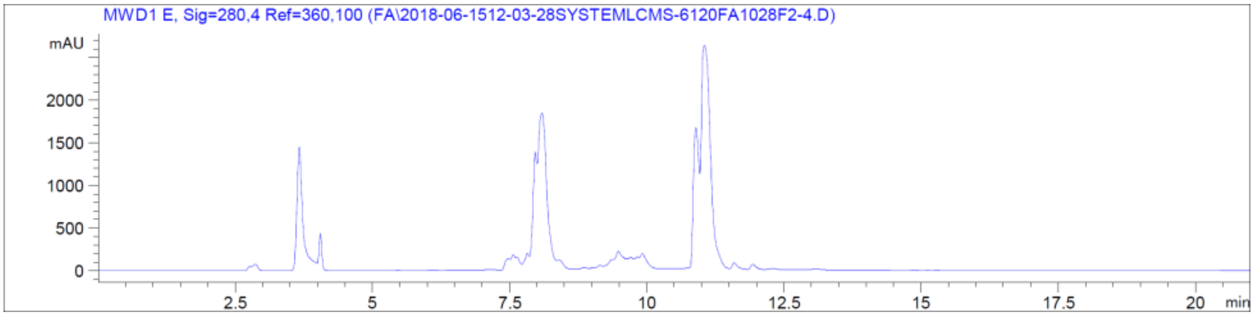

A

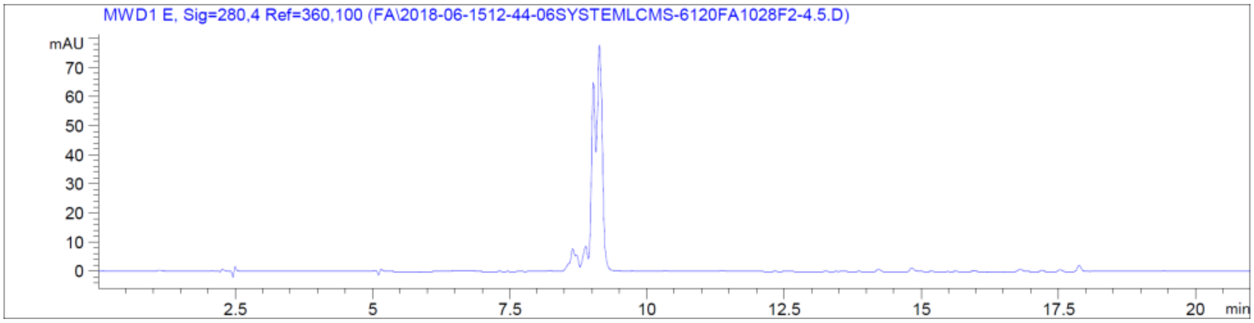

B

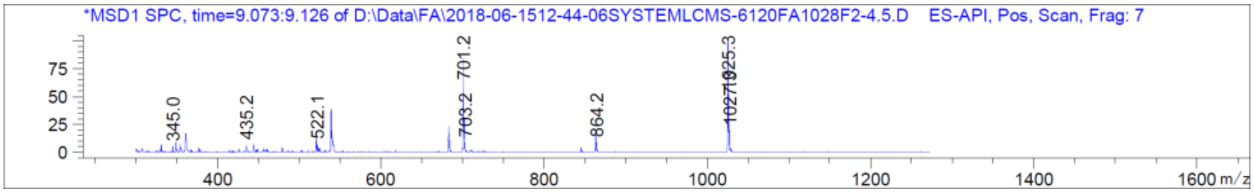

C

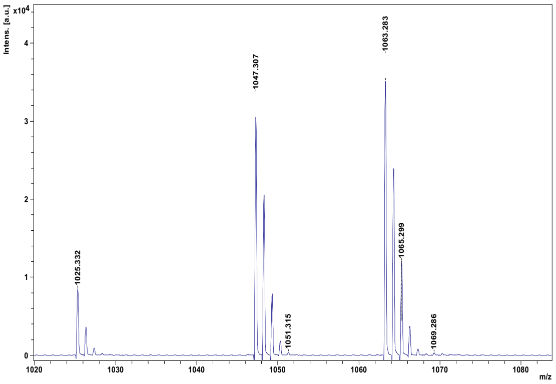

D

Spectra from the synthesis of NYST-F. A: HPLC spectrum of crude NYST-F. **B:** HPLC spectrum of purified NYST-F. **C:** LCMS spectrum of purified NYST-F [m/z (ESI) (M+H) $C_{45}H_{53}O_{27}$ calculated 1024.28, found 1025.33]. **D:** MALDI spectrum of purified NYST-F.

GAL-F

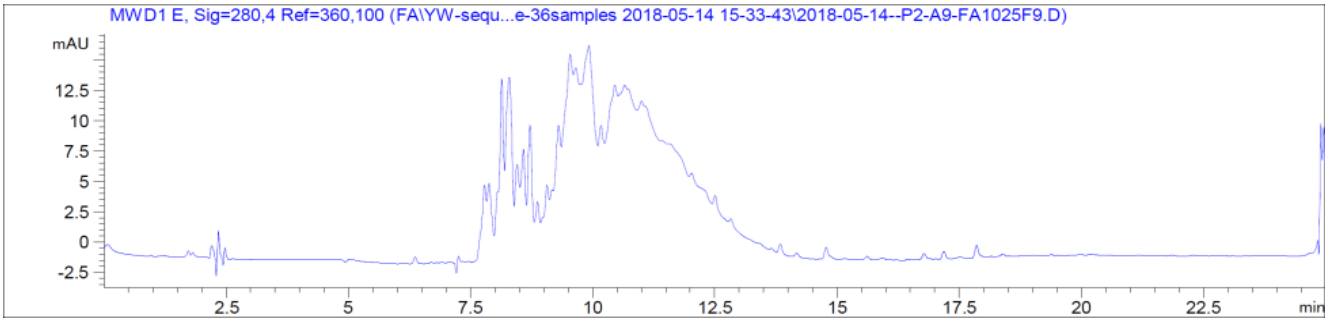

A

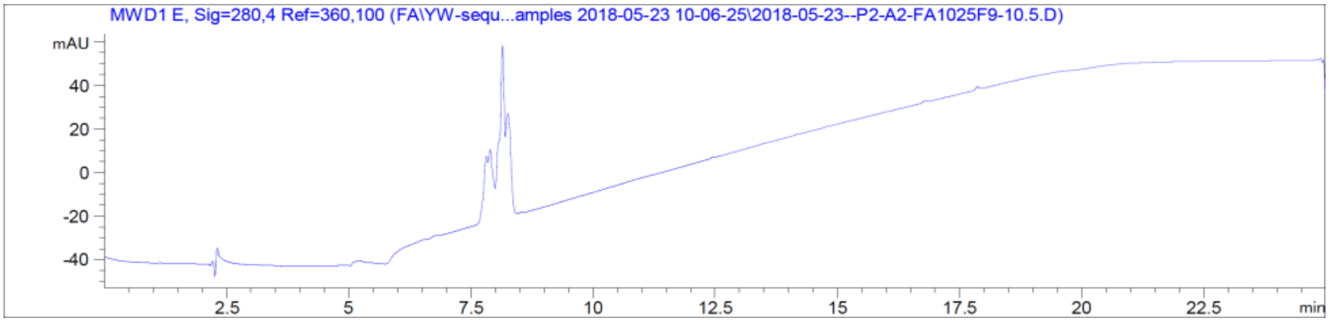

B

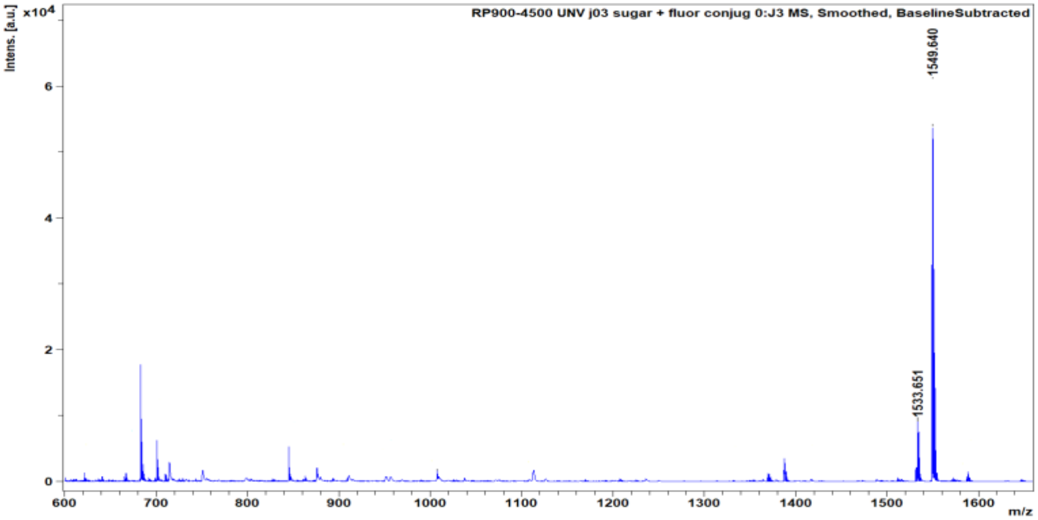

C

Spectra from the synthesis of GAL-F. A) HPLC spectrum of crude GAL-F. **B)** HPLC spectrum of purified GAL-F. **C)** MALDI spectrum of purified GAL-F [m/z (M+K) $C_{63}H_{82}O_{42}K$ calculated 1549.39, found 1549.64].

ARAB-F

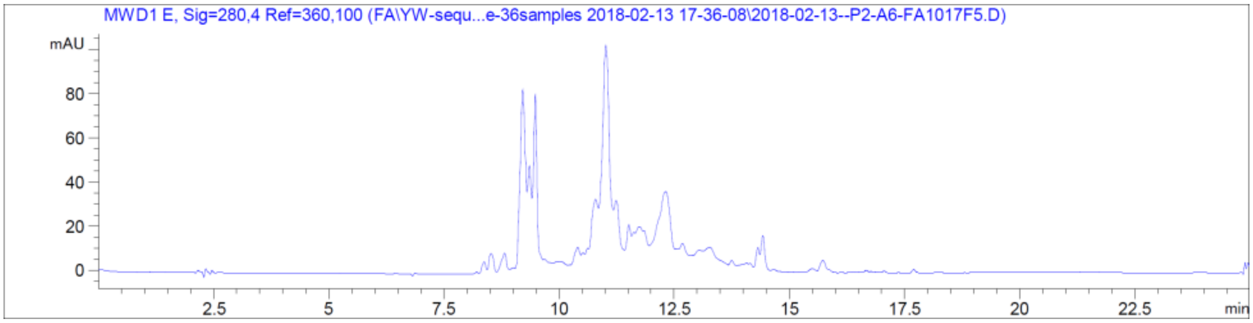

A

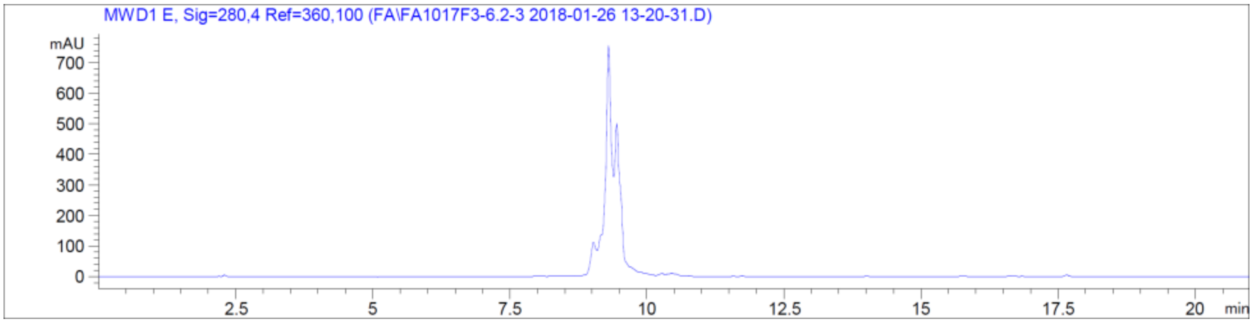

B

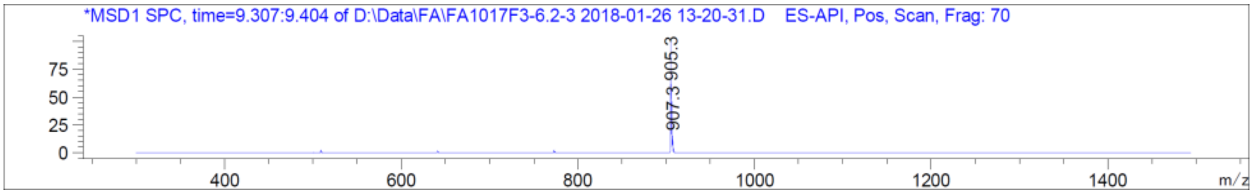

C

Spectra from the synthesis of ARAB-F. A) HPLC spectrum of crude ARAB-F. **B)** HPLC spectrum of purified ARAB-F. **C)** LCMS spectrum of purified ARAB-F [m/z (ESI) (M+H) $C_{41}H_{45}O_{23}$ calculated 905.23, found 905.3].

MANNO-F

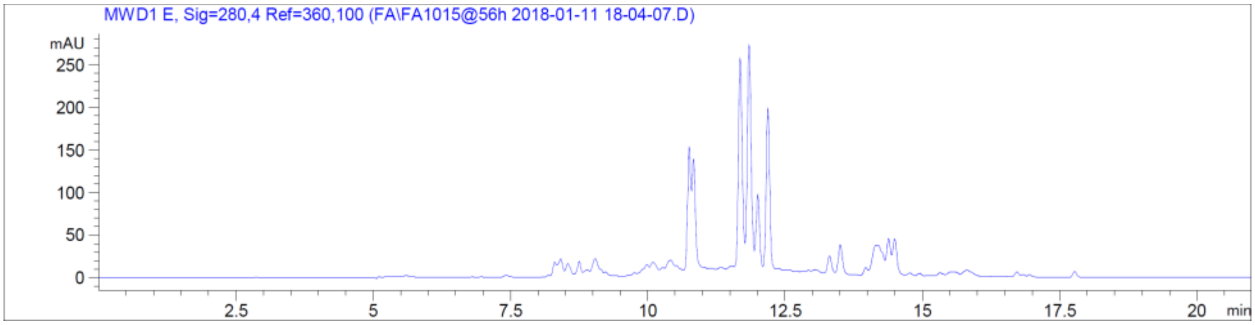

A

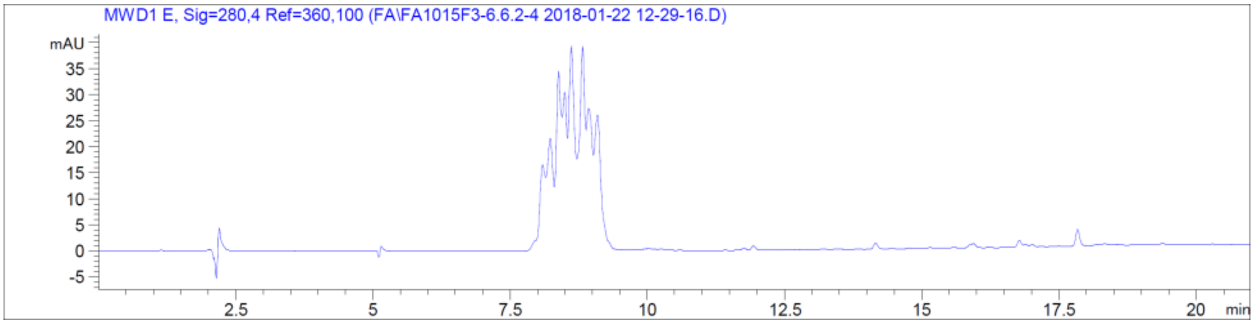

B

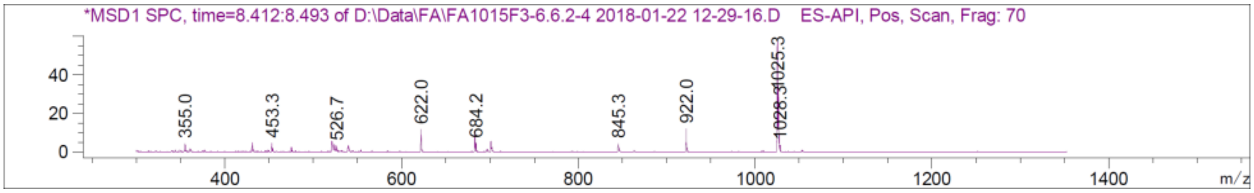

C

Spectra from the synthesis of MANNO-F. A) HPLC spectrum of crude MANNO-F. **B)** HPLC spectrum of purified MANNO-F. **C)** LCMS spectrum of purified MANNO-F [m/z (ESI) (M+H) $C_{45}H_{53}O_{27}$ calculated 1025.28, found 1025.3].

XYLO-F

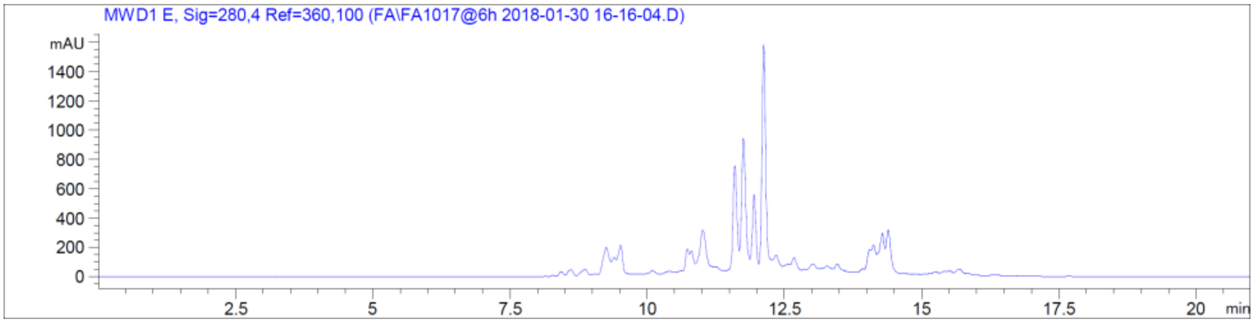

A

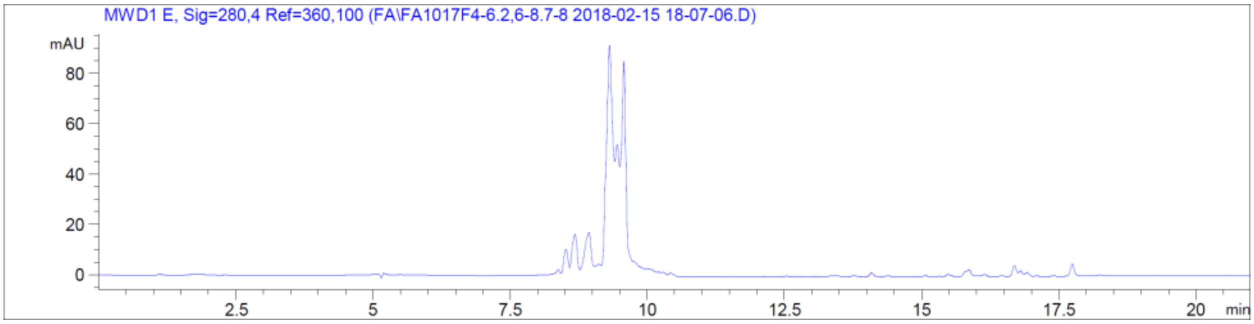

B

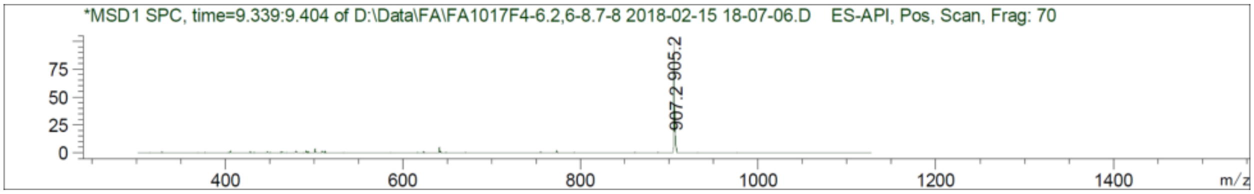

C

Spectra from the synthesis of XYLO-F. **A)** HPLC spectrum of crude XYLO-F. **B**) HPLC spectrum of purified XYLO-F. **C:** LCMS spectrum of purified XYLO-F [m/z (ESI) (M+H) $C_{41}H_{45}O_{23}$ calculated 905.23, found 905.2].

ARABXYLO-F

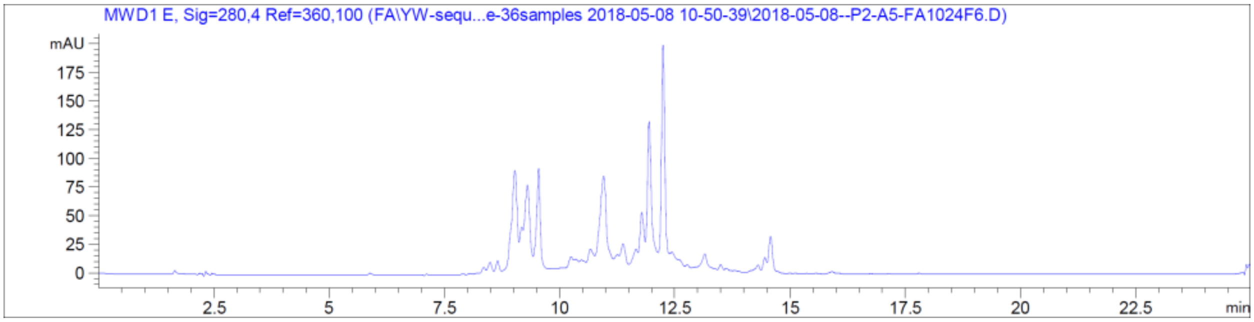

A

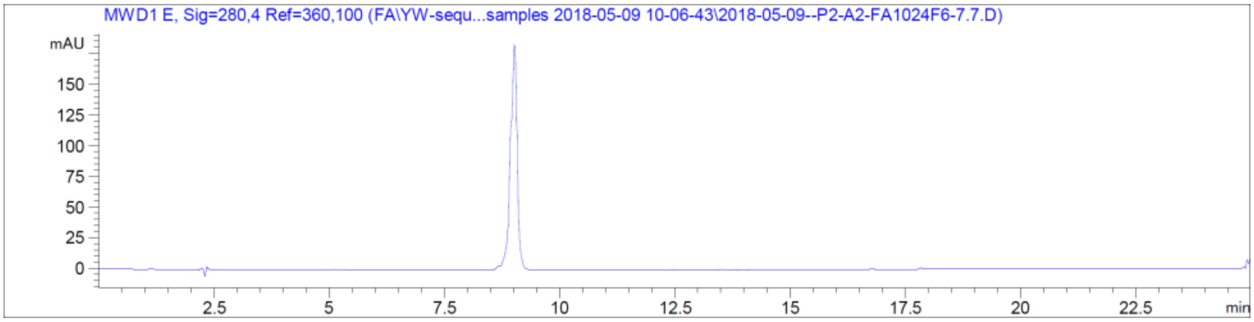

B

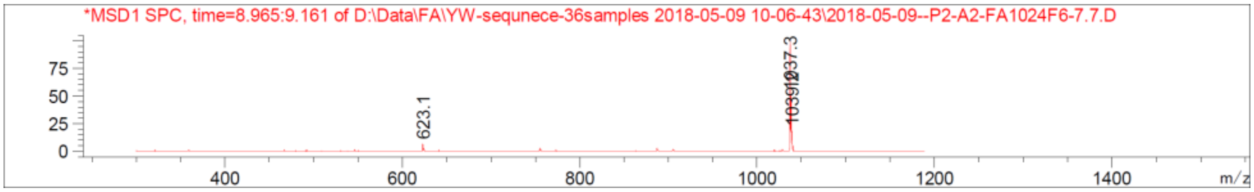

C

Spectra from the synthesis of ARABXYLO-F. A) HPLC spectrum of crude ARABXYLO-F. **B)** HPLC spectrum of purified ARABXYLO-F. **C)** LCMS spectrum of purified ARABXYLO-F [m/z (ESI) (M+H) $C_{46}H_{53}O_{27}$ calculated 1037.28, found 1037.3].

Glucose-F

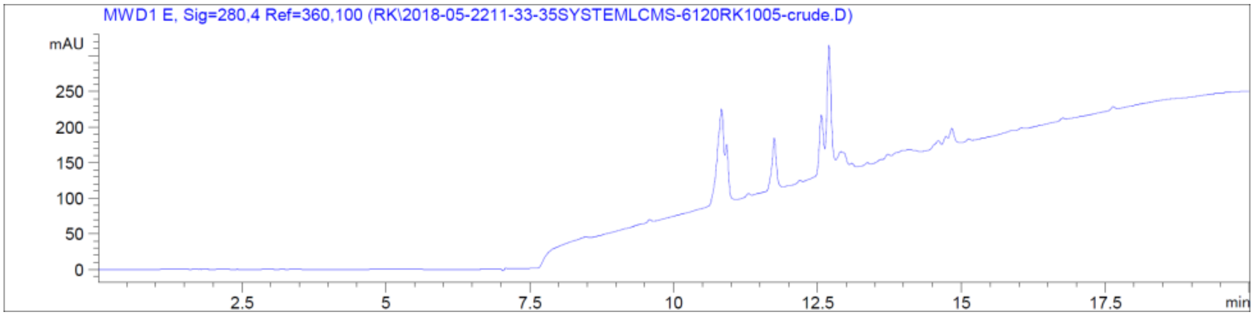

A

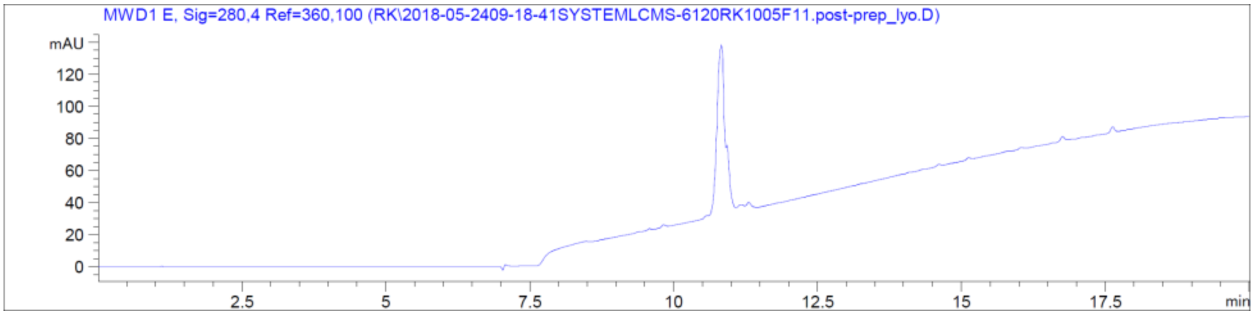

B

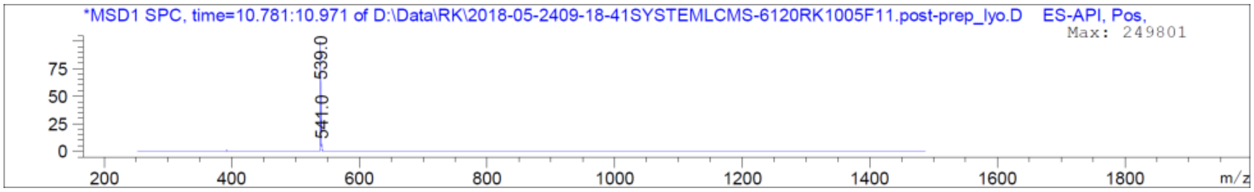

C

Spectra from the synthesis of glucose-F. A) HPLC spectrum of glucose-F. **B)** HPLC spectrum of purified glucose-F. **C)** LCMS spectrum of purified glucose-F [m/z (ESI) (M+H) $C_{27}H_{23}O_{12}$ calculated 539.12, found 539.0]

Maltose-F

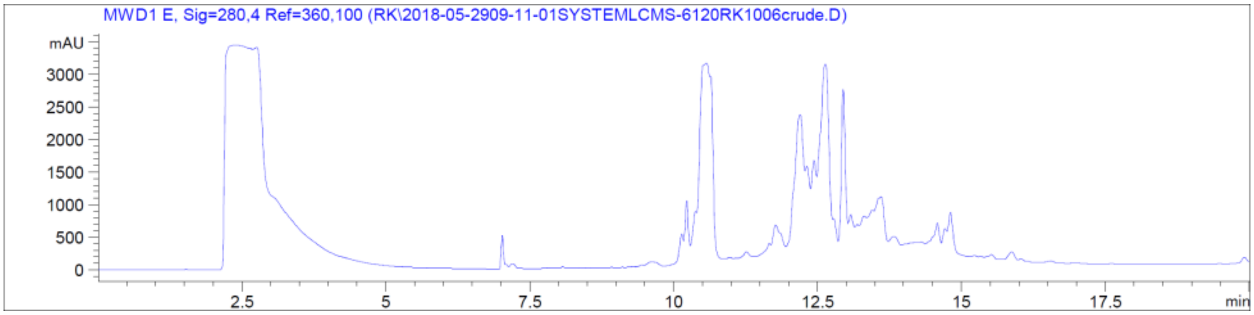

A

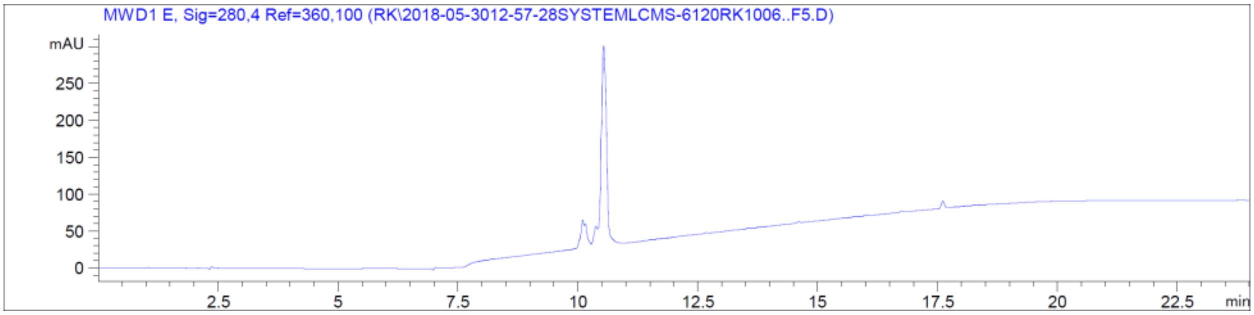

B

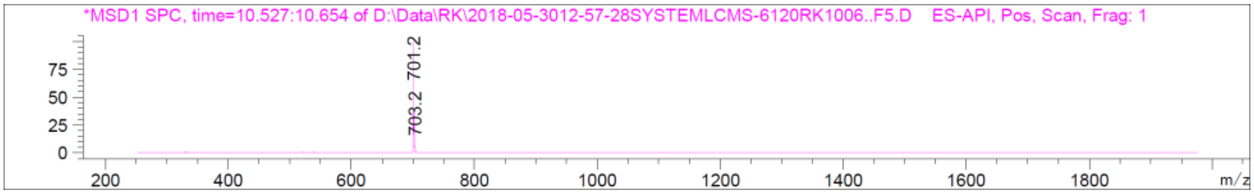

C

Spectra from the synthesis of maltose-F. A) HPLC spectrum of crude maltose-F. **B)** HPLC spectrum of purified maltose-F. **C)** LCMS spectrum of purified maltose-F [m/z (ESI) (M+H) $C_{33}H_{33}O_{17}$ calculated 701.17, found 701.2].

FOS-F

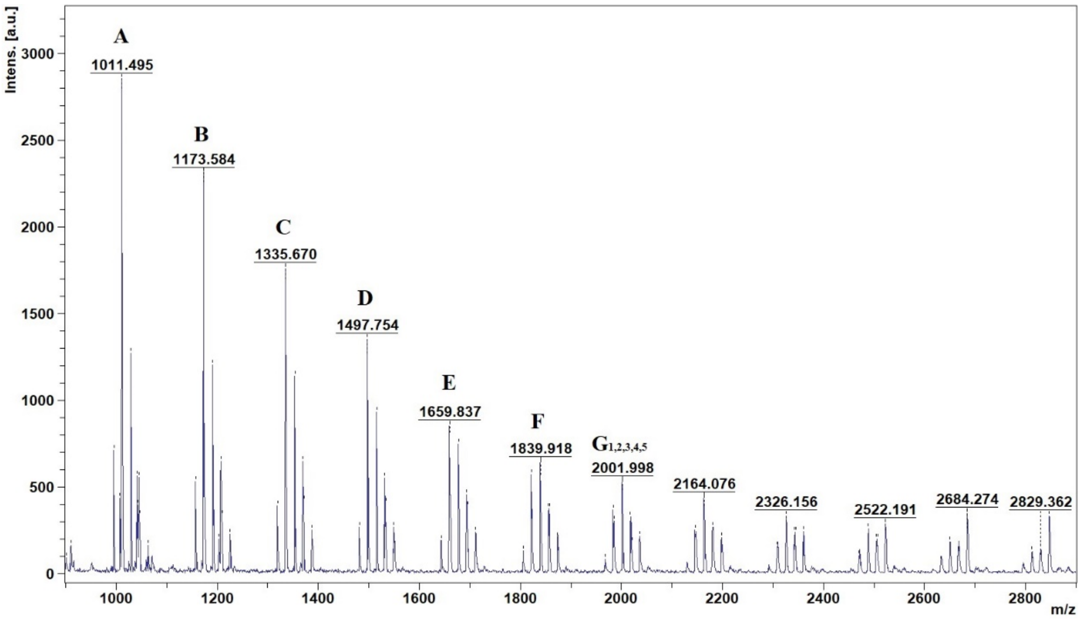

MALDI-TOF spectrum of FOS-F purified product. A: FOS_{16}-Fl [(M+3Na) $C_{117}H_{172}O_{87}Na_3^+$,calculated: 1012.813, found: 1011.495], **B:** FOS_{14} [(M+Na+K) $C_{84}H_{142}O_{71}$ Na^+ K^+, calculated: 1173.584 found: 1173.584], **C:** FOS_{16}[(M+Na+K) $C_{96}H_{162}O_{81}Na^+K^+$, calculated: 1336.480 found: 1335.670], **D:** FOS_{18} [(M+Na+K) $C_{108}H_{182}O_{91}Na^+$ K^+, calculated: 1498.540 found: 1497.754], **E:** FOS_{20} [(M+Na+K) $C_{120}H_{202}O_{101}Na^+$ K^+, calculated: 1660.6 found: 1659.837], **F:** FOS_{20}-Fl [(M+Na+K) $C_{141}H_{212}O_{107}Na^+$ K^+, calculated: 1839.84 found: 1839.918], **G1:** FOS_{24}[(M+Na+K) $C_{144}H_{242}O_{121}Na^+$ K^+, calculated: 1984.72 found: 1983.996],**G2:** FOS_{12} [(M+Na) $C_{72}H_{122}O_{61}Na^+$, calculated: 1985.720 found: 1986.061], **G3:** FOS_{12} [(M+K) $C_{72}H_{122}O_{61}K^+$, calculated: 2001.72 found: 2001.998], **G4:** FOS_{10}-Fl[(M+Na)$C_{81}H_{112}O_{57}Na^+$, calculated: 2020.08 found: 2020.981], **G5:** FOS_{10}-Fl [(M+K)$C_{81}H_{112}O_{57}K^+$, calculated: 2036.08 found: 2035.951]
