# Supplementary material for: Identifying glycan consumers in human gut microbiota samples using metabolic labeling coupled with fluorescence-activated cell sorting
Source: Nat Commun. 2023 Feb 7;14:662. doi: 10.1038/s41467-023-36365-8 (PMC9905522; doi:10.1038/s41467-023-36365-8)
Supplement: Supplementary file 2 — Description of Additional Supplementary Files [file 41467_2023_36365_MOESM2_ESM.docx]

**Description of Additional Supplementary Files**

**File Name**: Supplementary Data 1

**Description**: Sequences table for the 334 distinct ESVs identified using the ANCHOR pipeline.

**File Name**: Source Data

**Description**: All data from manuscript and supplementary information figures.
