# Supplementary material for: Identifying glycan consumers in human gut microbiota samples using metabolic labeling coupled with fluorescence-activated cell sorting
Source: Nat Commun. 2023 Feb 7;14:662. doi: 10.1038/s41467-023-36365-8 (PMC9905522; doi:10.1038/s41467-023-36365-8)
Supplement: Supplementary file 4 — Reporting Summary [file 41467_2023_36365_MOESM4_ESM.pdf]

nature portfolio

Corresponding author(s): Bastien Castagner

Last updated by author(s): Jan 20, 2023

Reporting Summary

Nature Portfolio wishes to improve the reproducibility of the work that we publish. This form provides structure for consistency and transparency in reporting. For further information on Nature Portfolio policies, see our Editorial Policies and the Editorial Policy Checklist.

Statistics

For all statistical analyses, confirm that the following items are present in the figure legend, table legend, main text, or Methods section.

n/a	Confirmed	
☐	☒	The exact sample size (*n*) for each experimental group/condition, given as a discrete number and unit of measurement
☐	☒	A statement on whether measurements were taken from distinct samples or whether the same sample was measured repeatedly
☐	☒	The statistical test(s) used AND whether they are one- or two-sided<br>*Only common tests should be described solely by name; describe more complex techniques in the Methods section.*
☒	☐	A description of all covariates tested
☐	☒	A description of any assumptions or corrections, such as tests of normality and adjustment for multiple comparisons
☐	☒	A full description of the statistical parameters including central tendency (e.g. means) or other basic estimates (e.g. regression coefficient) AND variation (e.g. standard deviation) or associated estimates of uncertainty (e.g. confidence intervals)
☐	☒	For null hypothesis testing, the test statistic (e.g. *F*, *t*, *r*) with confidence intervals, effect sizes, degrees of freedom and *P* value noted<br>*Give P values as exact values whenever suitable.*
☒	☐	For Bayesian analysis, information on the choice of priors and Markov chain Monte Carlo settings
☒	☐	For hierarchical and complex designs, identification of the appropriate level for tests and full reporting of outcomes
☒	☐	Estimates of effect sizes (e.g. Cohen's *d*, Pearson's *r*), indicating how they were calculated

Our web collection on statistics for biologists contains articles on many of the points above.

Software and code

Policy information about availability of computer code

Data collection	BD FACSDiva v8.0.2 for cytometry and Leica LAS X 3.5.7.23225 was used to collect images from microscope.
Data analysis	BD FACSDiva v8.0.2, FlowJo v10.8.1, Leica LAS X 3.5.7.23225, GraphPad Prism 9.5.0, ANCHOR v1.0, DESeq2 v1.38.3, MicrobiomeAnalystR v1.0

For manuscripts utilizing custom algorithms or software that are central to the research but not yet described in published literature, software must be made available to editors and reviewers. We strongly encourage code deposition in a community repository (e.g. GitHub). See the Nature Portfolio guidelines for submitting code & software for further information.

Data

Policy information about availability of data

All manuscripts must include a data availability statement. This statement should provide the following information, where applicable:

- Accession codes, unique identifiers, or web links for publicly available datasets
- A description of any restrictions on data availability
- For clinical datasets or third party data, please ensure that the statement adheres to our policy

The 16S sequencing data generated in this study have been deposited in the National Center for Biotechnology Information (NCBI)database under Bioproject accession code PRJNA925842 [https://www.ncbi.nlm.nih.gov/bioproject/925842]. The sequences for isolated bacteria reported in this paper have been deposited in the NCBI GenBank database under accession numbers: B. xylanisolvens CLD22001 (OP510057 [https://www.ncbi.nlm.nih.gov/nuccore/OP510057.1]), B. angulatum CLD22003 (OP512543 [https://www.ncbi.nlm.nih.gov/nuccore/OP512543]), B. caccae CLD22004 (OP512560 [https://www.ncbi.nlm.nih.gov/nuccore/OP512560]), B. uniformis CLD22005 (OP514970 [https://www.ncbi.nlm.nih.gov/nuccore/OP514970]), and E. coli CLD22006 (OP514723 [https://www.ncbi.nlm.nih.gov/nuccore/

OP514723]). The ANCHOR ESV sequences are available as a separate file (ANCHOR ESV sequences). Source data and statistical details are provided as a Source Data file.

Human research participants

Policy information about studies involving human research participants and Sex and Gender in Research.

Reporting on sex and gender	The microbiota samples used in this study were to provide a diversity of human gut bacteria. While there are sex differences in the human microbiota and its metabolism, the individual bacteria metabolism was studied here. Nevertheless, stool samples were collected from both sexes.
Population characteristics	Fresh fecal samples were collected from three healthy unrelated adult donors. 1 male of 30 years old with BMI of 26.9; 1 female of 34 years old with BMI of 26.3 and 1 male of 43 years old with BMI of 19.3.
Recruitment	Healthy donors were recruited by local advertisement. Subject information was recorded at the time of sampling. Only 3 samples were used as a collection of gut bacteria. Our conclusions strictly concern individual gut bacteria ability to consume glycans and are not extrapolated to a population.
Ethics oversight	The protocol A04-M27-15B was approved by the McGill Faculty of Medicine Institutional Review Board.

Note that full information on the approval of the study protocol must also be provided in the manuscript.

Field-specific reporting

Please select the one below that is the best fit for your research. If you are not sure, read the appropriate sections before making your selection.

☒ Life sciences ☐ Behavioural & social sciences ☐ Ecological, evolutionary & environmental sciences

For a reference copy of the document with all sections, see nature.com/documents/nr-reporting-summary-flat.pdf

Life sciences study design

All studies must disclose on these points even when the disclosure is negative.

Sample size	No statistical method was used to predetermine sample size.
Data exclusions	No data were excluded from the analyses.
Replication	All replicates are independent biological replicates with a minimum of n = 3.
Randomization	Randomization was not performed and was not necessary in our study.
Blinding	Blinding is not relevant for our study.

Reporting for specific materials, systems and methods

We require information from authors about some types of materials, experimental systems and methods used in many studies. Here, indicate whether each material, system or method listed is relevant to your study. If you are not sure if a list item applies to your research, read the appropriate section before selecting a response.

Materials & experimental systems

n/a	Involved in the study	
☒	☐	Antibodies
☒	☐	Eukaryotic cell lines
☒	☐	Palaeontology and archaeology
☒	☐	Animals and other organisms
☒	☐	Clinical data
☒	☐	Dual use research of concern

Methods

n/a	Involved in the study	
☒	☐	ChIP-seq
☐	☒	Flow cytometry
☒	☐	MRI-based neuroimaging

Flow Cytometry

Plots

Confirm that:

- [x] The axis labels state the marker and fluorochrome used (e.g. CD4-FITC).
- [x] The axis scales are clearly visible. Include numbers along axes only for bottom left plot of group (a 'group' is an analysis of identical markers).
- [x] All plots are contour plots with outliers or pseudocolor plots.
- [x] A numerical value for number of cells or percentage (with statistics) is provided.

Methodology

Sample preparation	The labeled bacteria are resuspended in PBS at the appropriate dilution to be further analyzed
Instrument	LSR Fortessa for cytometry and FACSAria-III for sorting (BD Biosciences)
Software	BD FACSDiva (BD Biosciences) and FlowJo (FlowJo LLC), analysis software was used for data analysis.
Cell population abundance	50,000 or 100 000 events per sample were analyzed to deteremined the labelled population and 1 to 3 million bacteria were sorted.
Gating strategy	To set the thresholds to specifically detect the cells labelled by the fluorescent probes, we used unlabeled cells as negative controls. The detection threshold for signals in the FITC channel was set at 10^3 compared to the maximum signal generated by<br>the negative control at 3·10^2. The gating threshold may be adjusted at different levels to adjust the sensitivity of the assay depending the probes.

- [x] Tick this box to confirm that a figure exemplifying the gating strategy is provided in the Supplementary Information.
